# Effectiveness of a long-term acupuncture treatment in patients with COPD: a randomised controlled trial

**DOI:** 10.1183/23120541.00668-2024

**Published:** 2025-05-19

**Authors:** Masao Suzuki, Yoshimitsu Takahashi, Daiki Inoue, Takamasa Kitajima, Tomonori Maekura, Keisuke Miki, Kiyomitsu Ikeoka, Miki Konishi, Ryoji Maekura, Motonari Fukui

**Affiliations:** 1 Respiratory Disease Center, Medical Research Institute, Kitano Hospital, PIIF, Tazuuke-Kofukai, Kita-ku, Japan; 2 Institute of Kampo Medical Research, Aizu Medical Center, Fukushima Medical University. Aizuwakamatsu City, Japan; 3 Department of Health Informatics, Kyoto University School of Public Health. Kyoto City, Japan; 4 Department of Respiratory Medicine, NHO Osaka Toneyama Medical Center, Toyonaka City, Japan; 5 Department of Family Medicine and Acupuncture Room Ikeoka Clinic, Jotoku, Japan

## Abstract

**Objective:**

We conducted a 1-year acupuncture intervention trial for patients with COPD and evaluated measures of quality of life (QOL).

**Methods:**

This 52-week randomised clinical trial was conducted in outpatient settings at two general hospitals and two clinics in Japan from January 2013 to October 2018. 45 patients were randomly assigned to the usual care group (UG) or the acupuncture treatment group (AG). Participants were patients with COPD classified as stage II or higher according to the Global Initiative for Chronic Obstructive Lung Disease classification. The UG received usual care in accordance with the guidelines, and the AG received acupuncture treatment once a week for 48 times in addition to usual care. Changes in outcome measures were compared using analysis of covariance, with the total domain score from the St George's Respiratory Questionnaire (SGRQ) measured at baseline and in week 52 as the primary outcome measure.

**Result:**

The 45 participants comprised 21 patients in the UG and 24 patients in the AG. In week 52, the SGRQ total domain score was significantly improved in the AG compared with the UG (mean±sd difference from baseline, −22.8±19.0 in the AG *versus* 6.8±13.5 in the UG; regression coefficient between groups: −26.9, 95% CI −35.8 to −17.9). No severe adverse events were reported.

**Conclusion:**

This study clearly suggested the long-term effectiveness of acupuncture. Acupuncture may offer a useful adjunctive therapy to improve QOL in COPD patients.

## Shareable abstract

**This study found that acupuncture in addition to conventional care is more effective than conventional care alone in improving the QOL of patients with stable COPD** https://bit.ly/3YJR1X1

## Introduction

COPD, a progressive disease associated with chronic inflammatory pulmonary syndrome, became the third leading cause of death worldwide in 2019 [1, 2]. As COPD progresses, physical, emotional and social functions are gradually affected and quality of life (QOL) declines. The most important factors contributing to reduced QOL include progression of dyspnoea, decreased ability to engage in physical activity and worsening of respiratory symptoms in association with exacerbations [3, 4]. Improving health status and QOL in COPD patients is thus one of the key treatment goals according to the Global Initiative for Chronic Obstructive Lung Disease (GOLD) management guidelines [5]. Although current pharmacological therapy may improve respiratory symptoms and QOL and reduce the frequency of exacerbations, the majority of COPD patients still suffer from severe symptoms, despite optimised drug combinations and innovative inhalers. According to the longitudinal analysis of the Swedish COPD national registry data reported by Sundh *et al*. [6], 74% of patients treated with triple therapy of a long-acting muscarinic antagonist, a long-acting β2 agonist and inhaled corticosteroids, as well as physical therapy, had persistent dyspnoea that interfered with activity, with a modified Medical Research Council (mMRC) score of ≥2, indicating that the current standard of care alone is insufficient. Recently, the Lancet Committee called for a complete reconsideration of the current approach to COPD [7]. In light of this situation, Janssens *et al*. [8] point out the importance of nonpharmacological therapy in the treatment strategy for COPD. On the other hand, in Japan, comprehensive rehabilitation other than pharmacological therapy is not available at any hospitals and clinics, so COPD patients cannot enjoy evidence-based nonpharmacological therapy.

It has been reported that about 4% (5 million people) of the Japanese population receive acupuncture treatment [9]. The percentage of those who use acupuncture treatment for respiratory symptoms is 1.2%, but the percentage of people who use acupuncture treatment for COPD is thought to be even lower. In Japan, acupuncture treatment is often used for chronic pain such as lower back pain, so neither patients nor doctors think of using acupuncture treatment for COPD.

Therefore, we conducted our preliminary research to develop a new nonpharmacological therapy for COPD. Our previous study examined short-term effects of acupuncture on patients with COPD, revealing improvements in dyspnoea on exertion (DOE) and QOL for acupuncture treatment compared with placebo acupuncture [10]. A systematic review/meta-analysis published after our study found statistically significant differences in dyspnoea, 6-min walk distance (6MWD), forced expiratory volume in 1 s (FEV_1_)%, St George's Respiratory Questionnaire (SGRQ) *etc*., between acupuncture treatment for COPD and the control group. However, these papers reported that caution is needed in interpreting the pooled results due to high heterogeneity among the studies [11, 12].

To date, there have been no studies examining long-term acupuncture intervention in chronic respiratory diseases, including COPD, and most have been short-term interventions, so it is unknown how long-term acupuncture intervention will affect the outcomes of respiratory symptoms. On the other hand, there are a number of studies examining the long-term effects of acupuncture intervention in chronic pain diseases, and some have reported that 6 months of acupuncture intervention showed improvement in pain over time compared with baseline, so it is thought that there is value in examining the long-term effects of intervention in COPD as well [13].

The present study was therefore undertaken as a randomised controlled trial to verify the long-term effects of acupuncture on patients with COPD in addition to conventional treatment by setting the treatment period to 1 year and using QOL as the primary end-point.

## Methods

### Study design and protocol

This study was a prospective investigator-initiated, multicentre, randomised, outcome assessor-blinded trial. The two-arm clinical trial was conducted in two general hospitals and two clinics in Japan between 17 July 2012 and 31 December 2022. The protocol and statistical analysis plan are provided in supplements 1 and 2, respectively. The institutional review boards of each general hospital approved the study. Written informed consent was obtained from each patient before baseline examination. The protocol of this study was registered as a clinical trial in the University Hospital Medical Information Network in Japan (UMIN000008934).

### Participants

Physicians in the departments of respiratory medicine of the general hospitals and clinics recruited participants from among outpatients seen during the patient entry period from 15 January 2013 to 31 October 2018. All participants met the following criteria: 1) COPD diagnosed by a physician; 2) ability to visit on an outpatient basis; 3) condition judged as stable condition by the physician; 4) COPD rated as grade 1 or more according to the mMRC criteria [14]; and 5) COPD classified as stage II or higher according to the GOLD criteria [15].

Patients presenting with signs and symptoms of heart failure (except for cor pulmonale), collagen disease, cancer or severe mental disorder, or patients undergoing pulmonary rehabilitation were excluded.

### Interventions

Acupuncture treatments were performed by four nationally licensed acupuncturists, each of whom had more than 10 years of clinical experience and had attended 5 weeks of centralised training before the study. Subjects in the usual care group (UG) were treated according to COPD guidelines. The acupuncture group (AG) received acupuncture treatment once a week 48 times during the year (52 weeks) in addition to the usual care. The acupuncture points utilised were those previously reported as effective for COPD in prior research [10, 16].

The acupuncture technique performed was Japanese style, using Japanese disposable needles that were 30–40 mm in length and 0.14–0.25 mm in thickness (Seirin Co., Shizuoka, Japan). World Health Organization standardised acupuncture points used in this study were as follows: LU1 (Zhongfu); LU9 (Taiyuan); CV4 (Guanyuan); CV12 (Zhongwan); CV17 (Zhanzhong); LI18 (Futu); KI3 (Taixi); ST36 (Zusanli); BL10 (Tianzhu); BL13 (Feishu); BL20 (Pishu); and BL23 (Shenshu) [17]. For bilateral acupuncture points, both were used.

After inserting an acupuncture needle into each acupuncture point and achieving deqi, the needle was manually rotated in one direction for 3 min. This was done for all acupuncture points, and total treatment time was approximately 60 min. Details of this method are provided in supplement 1.

### Outcomes

The primary outcome measure was the total domain score of the SGRQ measured at baseline and at the end of week 52. The SGRQ measures disease-specific QOL, with scores ranging from 0 to 100, with higher score indicating greater impairment of QOL [4, 18]. The SGRQ was administered using paper-format questionnaires completed by each patient in a hospital room with no research-related personnel present.

Secondary outcome measures included several items. First, the 12-point modified Borg scale (Dyspnoea MBS) [19] and lower limb fatigue (LLF) were assessed to measure the level of dyspnoea before and immediately after the 6-min walk test (6MWT) [20]. Also included were the 6MWD, minimum arterial oxygen saturation (*S*_pO_2__) per min, maximum heart rate per min and other SGRQ scores. The 6MWT was conducted using standard methods to assess exercise tolerance at baseline, week 12 and week 52.

Details regarding other outcome measures such as respiratory function parameters, nutritional status (body composition and haematological examination), muscle strength, thorax mobility, inflammation biomarkers, exacerbations, hospital visits and BODE (body mass index, airflow obstruction, dyspnoea and exercise) index are detailed in the full protocol (supplement 1).

Adverse events related to acupuncture treatment were evaluated by the acupuncturist in charge, who investigated the type and frequency of the adverse events each time acupuncture treatment was administered. To conceal allocation, adverse events were disclosed to the research team after the completion of statistical analysis, except for serious adverse events such as pneumothorax and neurological disorders.

All measurements were made at baseline and at the end of week 12 and week 52. Patients in the AG always received their final evaluation 1 week after their last acupuncture treatment.

All evaluations were performed by independent evaluators not involved in the research.

### Randomisation and blinding

#### Sequence generation and implementation

After the baseline assessment, an independent investigator assigned eligible patients to the UG or AG in a 1:1 ratio using a stratified (by GOLD classification II, III or IV), computer-generated random allocation sequence with a permutated block design and a block size of four.

#### Blinding

Block size and allocation sequence were concealed by the investigator until the end of the study. Physicians, outcome evaluators,and statisticians were all blinded to group assignments. In principle, acupuncturists were not allowed to disclose the allocation results to any study participants unless a patient experienced a serious adverse event, and the results were kept confidential until statistical analysis was completed. Also, patients were instructed not to report their allocation to their attending physicians or outcome assessors. Furthermore, no patient data were revealed to anyone involved in the study until all statistical analyses were completed. Acupuncture rooms in all facilities were completely private and located in a separate building from the hospital or clinic. As a result, there was no contact between participants and physicians, or between participants themselves around the acupuncture rooms. All data were entered by independent data entry personnel not involved in the study.

### Statistical analyses

The sample size was determined using a two-sided test of difference in the mean value of the difference of total domain SGRQ scores between baseline and week 12 of acupuncture treatment from our previous study [10]. Considering the minimal clinically important difference (MCID) of the total domain SGRQ score as 4 [21], and setting the absolute value of the difference to be detected as 10, sd as 10.0, significance level as 0.05 and power as 0.8, the sample size was calculated to be 34. Since this trial was considering clinical effects over the course of 1 year, a certain number of dropouts was expected. Assuming a dropout rate of approximately 30% (10 cases), the required final sample size was set at 44.

The dataset for this study was divided into two analysis groups, with the full analysis set (FAS) as the primary group and per-protocol set (PPS) as the secondary group. FAS and PPS are defined as all assigned patients excluding dropouts, and PPS as patients who achieved 40 or more acupuncture treatments in a year, excluding dropouts.

All statistical analyses were independently conducted by a statistician (Y.T.). Details are provided in the full study protocol and statistical analysis plan (supplements 1 and 2).

For all outcome measures, we looked at the change in difference between baseline and week 12, as well as between baseline and week 52. For the primary outcome measure and secondary outcome measures, we compared changes using ANCOVA, using baseline values and GOLD classification as covariates and treatment group as the factor of interest. For changes in other outcome measures, independent sample t-tests were used. Continuous and ordinal variables are shown as mean±sd, with significant differences judged from 95% confidence intervals (CIs). Count variables are shown as the count and percentage, and a chi-squared test or Fisher's exact test was applied to determine p-values. For changes in arterial blood oxygen saturation and heart rate during the 6MWT, we used the generalised linear mixed-effects model to analyse the difference between the 12-week change and 52-week change. *Post hoc* sensitivity analysis was also conducted using ANCOVA with multiple imputation of missing values. All statistical calculations were performed using a significance level of 0.05 with a power of 0.8, and all main analyses were carried out using STATA-16 (StataCorp LLC, College Station, TX, USA).

## Results

### Patient characteristics and baseline characteristic

**FIGURE 1:**
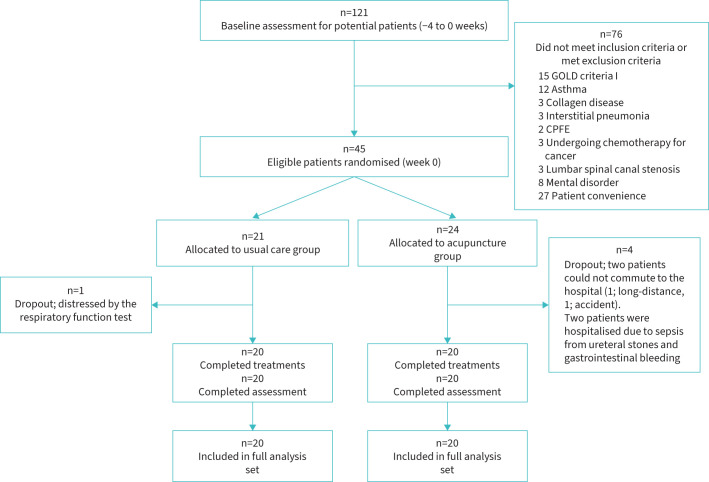
Flowchart showing trial group assignments, loss to follow-up, treatment completion and protocol deviations. GOLD: the Global Initiative for Chronic Obstructive Lung Disease; CPFE: combined pulmonary fibrosis and emphysema.

Between 8 January 2013 and 31 October 2018, a total of 121 patients were screened in the four sites. After excluding 76 patients, the remaining 45 patients were enrolled, comprising 24 patients in the AG and 21 patients in the UG. Figure 1 shows the trial profile.

Five participants failed to complete in the study. One UG participant dropped out due to distress during respiratory function testing before the week 12 exam. Two AG participants were unable to visit the hospital, one due to the distance to the hospital and the other due to a heavy rain disaster. Another two AG participants withdrew due to hospitalisation (one from sepsis caused by ureteral stones and the other from gastrointestinal bleeding).

All patients in the AG who dropped out did so before the week 12 evaluation. AG patients received an average of 45.2±1.4 acupuncture treatments per year, after excluding patients who dropped out. As a result, a total of 40 patients were included in the FAS: 20 patients in the AG (excluding 4 dropouts) and 20 patients in the UG (excluding 1 dropout).

The baseline characteristics of patients in each group, including medications taken by patients during the study, are shown in table 1.

**TABLE 1:** Baseline characteristics of randomised population

	UG (n=21)	AG (n=24)	Mean difference
**Sex (M/F)**	17/4	21/3	
**Age, years**	73.1±4.5	73.0±5.7	0.1
**mMRC**	2.9±0.8	2.7±1.0	−0.2
**Brinkman index**	1276.4±526.0	1181.3±674.1	95.2
**GOLD criteria**			
II	6	7	
III	11	11	
IV	4	6	
**Home oxygen therapy**	3	6	
**Medication**			
SABA	2	2	
LABA	9	7	
SAMA	1	1	
LAMA	15	13	
LAMA/LABA	6	9	
ICS	5	6	
ICS/LABA	11	7	
**SGRQ**			
Total domain	45.1±12.5	51.6±20.1	−6.6
Symptom domain	48.0±14.2	60.6±23.3	−12.6
Activity domain	62.4±15.8	64.6±20.6	−2.2
Impact domain	33.1±13.9	38.0±23.2	−4.9
**Exercise capacity**			
6MWT post-Dyspnoea Borg scale	5.6±2.4	5.5±1.9	0.2
6MWT post-limb fatigue Borg scale	4.6±3.1	4.6±2.1	−0.0
6MWD, m	372.7±126.4	385.3±112.6	−12.6
*S*_pO_2__, % lowest rate	86.4±5.1	89.0±4.6	−2.6
Pulse, bpm highest rate	108.9±15.1	114.3±17.3	−5.4
**Pulmonary function**			
VC, L	3.1±0.7	3.3±0.6	−0.2
IC, L	2.0±0.5	2.1±0.5	−0.1
FVC, L	3.0±0.7	3.2±0.7	−0.2
FEV_1_, L	1.2±0.5	1.2±0.5	0.0
FEV_1_ % predicted, %	46.4±15.5	43.7±16.8	2.7
**Expectations for acupuncture**			
I have high expectations	1	0	
Have medium expectations	1	1	
I'm expecting a little	4	3	
Indifferent	11	14	
I do not expect much	3	4	
I have no expectations	1	2	

Data are presented as mean±sd or n, unless indicated otherwise. UG: usual care group; AG: acupuncture group; mMRC: modified Medical Research Council Dyspnoea scale; GOLD: Global Initiative for Chronic Obstructive Lung Disease; SABA: short-acting β2 agonist; LABA: long-acting β2 agonists; SAMA: short-acting muscarinic antagonist; LAMA: long-acting muscarinic antagonist; ICS: inhaled corticosteroid; SGRQ: St George's Respiratory Questionnaire; 6MWT: 6-min walk test; 6MWD: 6-min walk distance; *S*_pO_2__: percutaneous oxygen saturation; bpm: beats per min; VC: vital capacity; IC: inspiratory capacity; FVC: forced vital capacity; FEV_1_: forced expiratory volume in 1 s.

### Primary outcome measure

**FIGURE 2:**
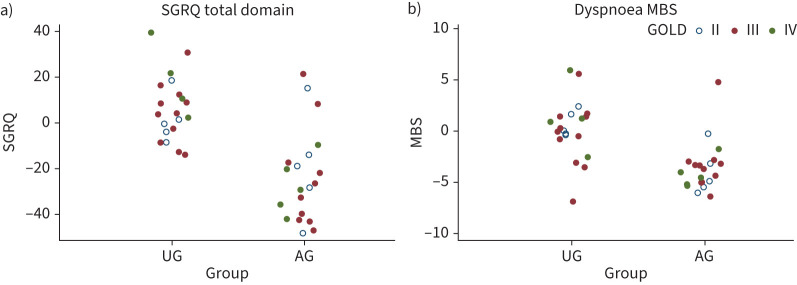
Differences in the St George's Respiratory Questionnaire (SGRQ) total domain and Dyspnoea modified Borg scale (MBS) in the usual care group (UG) and the acupuncture group (AG). Differences in a) the SGRQ total domain b) and Dyspnoea MBS between baseline and at week 52. Open blue circles indicate patients Global Initiative for Chronic Obstructive Lung Disease (GOLD) classification II; closed red circles indicate patients GOLD classification III; closed green circles indicate patients GOLD classification IV. Estimated regression coefficient (95% CI) were −26.9 (−35.8 to −17.9) and −4.1 (−5.4 to −2.7) for the SGRQ total domain and Dyspnoea MBS, ANCOVA, respectively.

The SGRQ total domain score improved from 50.1±18.0 at baseline to 27.3±10.1 at week 52 in the AG, whereas no improvement was seen in the UG (45.3±12.8 at baseline; 52.1±19.2 at week 52). The magnitude of the difference in SGRQ total domain score in the AG (−22.8±19.0) was significantly greater than that in the UG (6.8±13.5; mean difference −29.6, 95% CI −40.2 to −19.0; regression coefficient −26.9, 95% CI −35.8 to −17.9) with ANCOVA (table 2 and figure 2a).

**TABLE 2:** Changes in the primary and secondary outcome measure

	Baseline	After 12 weeks	After 52 weeks	CBA 12 weeks	MD (95% CI) RCE (95% CI)	CBA 52 weeks	MD (95% CI) RCE (95% CI)
**SGRQ** **total**							
UG	45.3±12.8	43.8±14.9	52.1±19.2	−1.5±7.2	−19.4 (−26.5 to −12.3)	6.8±13.5	−29.6 (−40.2 to −19.0)
AG	50.1±18.0	29.1±10.2	27.3±10.1	−20.9±13.9	−17.3 (−23.1 to −11.5)	−22.8±19.0	−26.9 (−35.8 to −17.9)
**Symptom**							
UG	48.1±14.6	50.9±22.1	51.5±17.6	2.8±21.3	−33.3 (−46.5 to −20.0)	3.4±10.8	−38.7 (−51.0 to −26.4)
AG	60.4±22.8	30.0±11.4	25.1±14.1	−30.4±20.2	−24.8 (−35.9 to −13.8)	−35.3±24.8	−30.5 (−40.3 to −20.7)
**Activity**							
UG	63.1±15.8	61.0±15.5	65.9±19.1	−2.1±10.3	−12.2 (−19.4 to −5.1)	2.7±13.6	−20.0 (−29.9 to −10.1)
AG	62.9±20.4	48.5±17.9	45.6±17.1	−14.4±12.0	−12.4 (−19.1 to −5.7)	−17.3±17.2	−20.5 (−29.5 to −11.5)
**Impact**							
UG	33.2±14.2	29.2±16.2	38.4±23.7	−4.0±10.6	−17.2 (−26.2 to −8.2)	5.2±20.2	−27.1 (−40.5 to −13.6)
AG	36.1±20.2	14.9±9.3	14.3±8.8	−21.2±16.9	−15.7 (−22.7 to −8.7)	−21.8±21.7	−25.5 (−36.6 to −14.5)
**6MWT** **Dyspnoea MBS, score**							
UG	5.7±2.5	5.9±2.8	6.0±2.7	0.3±2.7	−3.0 (−4.5 to −1.6)	0.3±2.6	−3.8 (−5.3 to −2.2)
AG	5.2±1.7	2.4±1.4	1.7±1.6	−2.8±1.7	−3.3 (−4.6 to −2.0)	−3.5±2.3	−4.1 (−5.4 to −2.7)
**LLF MBS, score**							
UG	4.6±3.2	5.0±3.0	5.3±3.1	0.4±2.0	−2.1 (−3.4 to −0.7)	0.7±2.5	−3.3 (−4.9 to −1.7)
AG	4.4±2.2	2.8±1.9	1.8±1.6	−1.6±2.3	−2.1 (−3.4 to −0.9)	−2.6±2.5	−3.5 (−4.8 to −2.2)
**6MWD, m**							
UG	366.2±126.0	352.0±129.0	331.0±137.0	−14.6±65.8	72.0 (36.0 to 108.0)	−35.4±83.8	112.0 (62.0 to 162.0)
AG	387.3±115.2	444.6±112.4	463.7±121.7	57.3±43.5	74.4 (38.0 to 110.8)	76.4±72.6	116.9 (66.5 to 167.2)
**Lowest *S*_pO_**_2_ **, %**							
UG	86.2±5.1	86.7±4.9	85.0±5.6	0.6±3.2	2.5 (0.5 to 4.4)	−1.2±3.3	5.2 (3.2 to 7.1)
AG	88.6±4.7	91.6±4.0	92.6±3.6	3.0±2.7	3.2 (1.3 to 5.0)	4.0±2.8	5.6 (3.6 to 7.6)
**Highest pulse, bpm**							
UG	108.5±15.3	111.0±18.1	116.5±15.9	2.7±13.8	−12.0 (−21.0 to −2.0)	8.0±16.1	−23.0 (−32.0 to −13.0)
AG	115.2±18.1	106.3±12.1	100.5±13.4	−9.0±16.4	−9.5 (−17.6 to −1.5)	−14.7±14.0	−20.3 (−28.1 to −12.5)

Data are presented as mean±sd unless otherwise specified. CBA: change from baseline to after; MD: mean difference; RCE: regression coefficient; UG: usual care group; AG: acupuncture group; SGRQ: St George's Respiratory Questionnaire; GOLD: Global Initiative for Chronic Obstructive Lung Disease; MBS: modified Borg scale; LLF MBS: lower limb fatigue modified Borg scale; 6MWD: 6-min walk distance; *S*_pO_2__: percutaneous oxygen saturation; bpm: beats per min.

Further analysis following multiple imputation showed a regression coefficient of −24.7. 95% CI −34.3 to −15.2 (table 3).

**TABLE 3:** Analysis of covariance after multiple imputation with *post hoc* analysis (primary and secondary outcome measures)

	Baseline	After 12 weeks	RCE (95% CI)	After 52 weeks	RCE (95% CI)
**SGRQ total**					
UG	45.3±2.9	43.3±3.3	−16.0	51.1±4.3	−24.7
AG	50.1±4.0	30.7±2.5	(−22.3 to −9.7)	29.2±3.1	(−34.3 to −15.2)
**Symptom**					
UG	48.2±3.3	50.5±4.9	−28.3	50.5±4.1	−32.5
AG	60.4±5.1	31.9±3.2	(−41.6 to −15.1)	27.4±3.7	(−44.9 to −20.1)
**Activity**					
UG	63.1±3.5	60.5±3.4	−10.5	64.7±4.3	−17.5
AG	62.6±4.6	49.9 ±3.7	(−17.4 to −3.7)	47.0±3.8	(−26.9 to −8.3)
**Impact**					
UG	33.2±3.2	28.7±3.5	−13.6	37.5±5.2	−22.1
AG	36.1±4.5	16.1±2.3	(−21.5 to −5.7)	15.9±2.8	(−33.6 to −10.6)
**6MWT Dyspnoea MBS, score**					
UG	5.7±0.6	5.8±0.6	−3.0	5.8±0.6	−3.5
AG	5.2±0.4	2.7±0.4	(−4.6 to −1.3)	2.1±0.5	(−5.2 to −1.8)
**LLF MBS, score**					
UG	4.6±0.7	4.9±0.7	−2.0	5.2±0.7	−3.3
AG	4.4±0.5	3.0±0.5	(−3.5 to −0.6)	2.0±0.5	(−4.9 to −1.6)
**6MWD, m**					
UG	366.2±28.2	356.2±28.5	69.4	336.6±30.8	108.2
AG	387.3±25.8	435.8±24.2	(24.9 to 113.8)	457.6±28.4	(50.2 to 116.1)
**Lowest *S*_pO_**_2_ **, %**					
UG	86.2±1.1	86.9±1.1	1.8	85.2±1.7	4.0
AG	88.6±1.1	91.1±1.0	(−0.6 to 4.2)	92.0±1.1	(1.3 to 6.8)
**Highest pulse, bpm**					
UG	108.5±3.4	111.0±4.0	−11.2	116.2±3.6	−21.8
AG	115.2±4.1	107.1±3.0	(−20.8 to −1.6)	101.8±3.3	(−31.8 to −11.8)

Data are presented as mean±se unless otherwise specified. The number of cases after multiple imputation was 21 for UG and 24 for AG. CBA: change from baseline to after; RCE: regression coefficient; AG: acupuncture group; UG: usual care group; SGRQ: St George's Respiratory Questionnaire; MBS: modified Borg scale; LLF: lower limb fatigue; 6MWD: 6-min walk distance; *S*_pO_2__: percutaneous oxygen saturation; bpm: beats per min.

### Secondary outcome measures

Dyspnoea MBS after 6MWT in the AG improved from baseline to both week 12 and week 52, but no improvement was apparent in the UG (mean difference −3.0, 95% CI −4.5 to −1.6 and −3.8, 95% CI −5.3 to −2.2, respectively; regression coefficient −3.3, 95% CI −4.6 to −2.0 and −4.1, 95% CI −5.4 to −2.7, respectively) (table 2 and figure 2b).

The results of linear mixed-effects model analysis for changes in *S*_pO_2__ before and during 6MWT also showed significant differences (2.3%, 95% CI 1.3 to 3.3 at rest and 0.6%, 95% CI 0.2 to 1.0 per min during 6MWT) between the two groups at week 52. Details of these results are shown in supplement 3.

At week 12, the difference in SGRQ total domain score was significantly greater in the AG than in the UG (mean difference −19.4, 95% CI −26.5 to −12.3; regression coefficient −17.3, 95% CI −23.1 to −11.5 with ANCOVA). Subdomains of the SGRQ also showed significant improvements in the AG compared with the UG at both week 12 and week 52 compared with baseline (table 2).

### Other outcome measures

#### Respiratory function

At week 52, all respiratory function evaluations except inspiratory capacity showed significant improvements in the AG compared with the UG (table 4).

**TABLE 4:** Changes in the respiratory function parameters

Respiratory function	Baseline	After 12 weeks	After 52 weeks	CBA 12 weeks	MD (95% CI)	CBA 52 weeks	MD (95% CI)
**VC, L**							
UG	3.0±0.7	3.1±0.7	3.0±0.7	0.1±0.2	−0.1	−0.1±0.4	0.2
AG	3.2±0.7	3.2±0.7	3.4±0.6	0.0±0.2	(−0.2 to 0.0)	0.2±0.3	(0.0 to 0.5)
**IC, L**							
UG	1.9±0.5	2.0±0.6	1.9 ±0.5	0.1 ±0.2	−0.1	−0.1±0.4	0.1
AG	2.1±0.5	2.1±0.5	2.1±0.4	0.0±0.3	(−0.3 to 0.0)	0.0±0.3	(−0.1 to 0.3)
**FVC, L**							
UG	3.0 ±0.7	3.1±0.7	2.9±0.7	0.1±0.1	−0.8	−0.1±0.4	0.3
AG	3.1±0.7	3.1±0.6	3.3±0.6	0.0±0.2	(−0.2 to 0.0)	0.2±0.2	(0.1 to 0.5)
**FEV_1_, L**							
UG	1.2±0.5	1.2±0.5	1.1±0.5	0.0±0.1	0.0	−0.1±0.2	0.2
AG	1.1±0.1	1.1±0.4	1.2±0.4	0.0±0.1	(−0.1 to 0.0)	0.1±0.1	(0.1 to 0.3)
**FEV_1_% pred**							
UG	45.9±15.7	46.5±16.2	42.9±16.9	0.6±3.0	−0.7	−3.0±5.0	6.3
AG	41.9±17.0	41.8±16.8	45.2±19.5	−0.1±3.1	(−2.6 to 1.3)	3.3±5.3	(3.0 to 9.6)
**FRC, L**							
UG	3.4±0.7	3.6±0.9	3.6±1.0	0.2±0.4	−0.2	0.2 ±0.5	−0.5
AG	4.1±0.8	4.0±0.7	3.8±0.9	−0.1±0.3	(−0.5 to −0.0)	−0.3±0.6	(−0.9 to −0.1)
**RV, L**							
UG	2.3±0.6	2.4±0.7	2.6±0.8	0.1±0.4	−0.2	0.3±0.6	−0.6
AG	3.0±0.6	2.8±0.6	2.6±0.7	−0.1±0.3	(−0.5 to −0.0)	−0.3±0.7	(−1.0 to −0.2)
**TLC, L**							
UG	5.4±1.0	5.6±1.2	5.6±1.0	0.2±0.4	−0.3	0.2±0.3	−0.5
AG	6.3±0.9	6.2±0.9	6.1±1.0	−0.2±0.4	(−0.6 to −0.1)	−0.3±0.7	(−0.8 to −0.1)
**RV/TLC**							
UG	42.7±7.6	43.3±6.6	45.8±9.7	0.6±4.3	−1.6	3.1±8.6	−6.8
AG	46.8±7.4	45.9±8.4	43.1±7.9	−0.9±4.7	(−4.4 to 1.3)	−3.7±7.4	(−12.0 to −1.7)
***D*_LCO_, mL·min^−1^·mmHg^−1^**							
UG	11.1±3.6	11.8±4.3	10.8±4.1	0.8±1.4	−0.1	−0.2±1.4	1.7
AG	10.7±3.9	11.4±4.2	12.1±4.1	0.7±1.5	(−1.0 to 0.9)	1.4±1.4	(0.8 to 2.6)
***D*_LCO_, %**							
UG	75.6±23.0	81.2±26.4	76.4±28.4	5.6±8.4	−1.1	0.9±9.6	8.2
AG	66.0±21.1	70.6±22.3	75.1±21.6	4.5±10.0	(−7.0 to 4.8)	9.1±9.0	(2.3 to 14.1)
***D*_LCO_/*V*_A_, mL·min^−1^·mmHg^−1^·L^−1^**							
UG	2.6±0.8	2.7±0.9	2.5±0.9	0.1±0.2	0.0	−0.1±0.3	0.3
AG	2.3±0.9	2.4±1.0	2.5±1.0	0.1±0.2	(−0.2 to 0.1)	0.2±0.3	(0.1 to 0.5)

Data are presented as mean±sd unless otherwise specified. CBA: change from baseline to after; MD: mean difference; UG: usual care group; AG: acupuncture group; VC: vital capacity; IC: inspiratory capacity; FVC: forced vital capacity; FEV_1_: forced expiratory volume in 1 s; FEV_1_% pred: per cent predicted forced expiratory volume in 1 s; FRC: functional residual capacity; RV: residual volume; TLC: total lung capacity; *D*_LCO_: diffusing capacity of the lung for carbon monoxide; *V*_A_: alveolar volume.

#### Measurements of nutritional status, muscle strength and thorax mobility

Significant improvements at week 12 and week 52 in nutritional status of body composition, haematological examinations (pre-albumin, albumin and haemoglobin), muscle strength and thorax mobility were found in the AG compared with the UG (table 5 and supplement 3).

**TABLE 5:** Changes in the nutritional status

Nutrition status	Baseline	After 12 weeks	After 52 weeks	CBA 12 weeks	MD (95% CI)	CBA 52 weeks	MD (95% CI)
**Body composition**							
**BMI, kg·m^−2^**							
UG	22.0±3.2	21.9±3.2	21.2±3.2	−0.1±0.6	0.7	−0.8±0.7	1.7
AG	21.9±3.3	22.5±3.1	22.8±3.3	0.6±0.5	(0.3 to 1.1)	0.9±0.8	(1.2 to 2.2)
**TSF, mm**							
UG	12.1±5.4	10.5±5.3	10.2±4.7	−1.6±2.8	5.8	−1.9±3.2	9.9
AG	14.1±4.5	18.3±3.4	22.1±4.4	4.2±4.0	(3.6 to 7.9)	8.0±3.9	(7.5 to 12.2)
**MAC, cm**							
UG	25.6±2.6	25.0±2.4	24.4±2.7	−0.6±1.6	3.4	−1.3±1.9	5.8
AG	25.1±3.2	27.9±3.5	29.7±3.9	2.8±2.5	(2.0 to 4.7)	4.6±3.4	(4.1 to 7.6)
**AMC, cm**							
UG	21.8±2.5	21.7±2.2	21.1±2.1	−0.1±1.5	1.6	−0.7±1.7	2.7
AG	20.7±2.9	22.1±3.5	22.7±4.1	1.4±2.5	(0.2 to 2.9)	2.0±3.8	(0.8 to 4.6)
**AMA, cm^2^**							
UG	38.4±8.2	37.9±7.5	35.9±7.0	−0.5±5.0	5.7	−2.5±5.3	10.1
AG	34.7±10.8	40.0±13.8	42.4±16.1	5.2±9.3	(0.9 to 10.5)	7.7±14.7	(3.1 to 17.2)
**Haematological examination**							
**Prealbumin, mg·dL^−1^**							
UG	26.9±5.1	26.4±6.4	26.0±5.6	−0.5±3.1	2.6	−0.9±3.3	5.4
AG	25.4±4.2	27.5±4.2	29.8±4.2	2.1±3.2	(0.6 to 4.6)	4.5±3.2	(3.3 to 7.5)
**Albumin, mg·dL^−1^**							
UG	4.4±0.3	4.3±0.3	4.2±0.4	−0.0±0.2	0.3	−0.2±0.4	0.8
AG	4.1±0.5	4.4±0.3	4.7±0.4	0.3±0.5	(0.1 to 0.6)	0.6±0.6	(0.5 to 1.1)
**Haemoglobin, g·dL^−1^**							
UG	14.7±1.8	14.3±1.9	13.8±2.2	−0.4±1.2	0.9	−0.9±1.5	1.6
AG	14.2±1.4	14.7±1.3	14.9±1.3	0.5±0.6	(0.4 to 1.5)	0.7±1.0	(0.8 to 2.4)

Data are presented as mean±sd unless otherwise specified. CBA: change from baseline to after; MD: mean difference; UG: usual care group; AG: acupuncture group; BMI: body mass index; TSF: triceps skinfold thickness; MAC: mid-upper arm circumference; AMC: arm muscle circumference; AMA: arm muscle area.

#### Biomarkers of inflammation

At weeks 12 and 52, improvements in interleukin (IL)-6, tumour necrosis factor (TNF)-α and high-sensitivity C-reactive protein (hs-CRP) were found in the AG, displaying significant reductions in scores compared with the UG (table 6).

**TABLE 6:** Changes in inflammation biomarkers

	Baseline	After 12 weeks	After 52 weeks	CBA 12 weeks	MD (95% CI)	CBA 52 weeks	MD (95% CI)
**IL-6, pg·mL^−1^**							
UG	1.9±0.9	2.0±0.8	2.6±1.3	0.2±0.4	−1.8	0.7±1.2	−3.0
AG	3.3±2.1	1.7±0.8	1.1±0.4	−1.7±1.7	(−2.6 to −1.0)	−2.3±2.0	(−4.1 to −1.9)
**TNF-α, pg·mL^−1^**							
UG	1.6±0.9	1.9±0.9	2.6±1.5	0.4±0.6	−2.4	1.0±1.4	−3.5
AG	3.4±2.6	1.4±0.5	0.8±0.5	−2.0±2.7	(−3.6 to −1.1)	−2.5±2.5	(−4.8 to −2.3)
**Hs-CRP, ng·mL^−1^**							
UG	1481.0±1095.2	1878.5±1554.4	2350.9±2181.1	397.5±1074.3	−3012.1	869.9±1852.8	−4202.2
AG	4051.6±3139.4	1437.0±1328.8	719.3±526.2	−2614.6±2417.5	(−4209.0 to −1815.0)	−3332.3±2754.2	(−5705.0 to −2700.0)

Data are presented as mean±sd unless otherwise specified. CBA: change from baseline to after; MD: mean difference; UG: usual care group; AG: acupuncture group; IL: interleukin; TNF: tumour necrosis factor; Hs-CRP: high-sensitivity C-reactive protein.

#### Exacerbations and hospital visits

During the 52-week study period, the number of moderate or high exacerbations was significantly lower in the AG than in the UG, and the number of unscheduled hospital visits associated with exacerbation was also significantly lower. However, no significant differences were seen in the number of hospitalisations associated with exacerbation (UG, 3 patients; AG, 0 patients) (supplement 3).

#### BODE index

The difference in BODE index in the AG was significantly greater than that in the UG (mean difference −2.7, 95% CI −3.4 to −1.9 at week 52) (supplement 3).

### Adverse reactions

Minor adverse reactions associated with acupuncture reported by patients during the study were fatigue (five patients), small subcutaneous haemorrhage (seven patients) and needle site pain (three patients). All events were minor reactions and the patients recovered quickly. No serious events due to acupuncture treatment were reported.

## Discussion

To the best of our knowledge, this represents the first long-term randomised study to demonstrate the efficacy of acupuncture treatment for improving QOL and exercise tolerance in COPD patients. Our main finding was that the difference in SGRQ total domain score at week 52 was −29.6 for AG compared with UG. In principle, placebo acupuncture has to be used for an appropriate control group. However, during the initial stage of research planning, the acupuncturists suggested that completing a 1-year intervention without patients being aware of the use of placebo acupuncture would be difficult, since acupuncture is a well-known treatment in Japan. We therefore decided to set two groups without using placebo acupuncture, with one receiving usual care only and the other receiving usual care plus acupuncture.

The primary end-point of this study was the change in SGRQ total domain score, representing a soft end-point. To enhance the robustness of the study results, we strictly masked group allocations to all study personnel except for patients and acupuncturists. We also set many hard end-points, including 6MWT, respiratory function tests and blood tests. The study results indicate that the AG achieved significant improvements in many of the hard end-points compared with the UG, supporting the robustness of the results. Furthermore, *post hoc* sensitivity analysis demonstrated a significant improvement in SGRQ change in the AG compared with the UG, confirming the robustness of the primary analysis. Nevertheless, the involvement of the placebo effect on the primary outcome needs to be further evaluated in future work.

The placebo effect in general offers strong evidence that patient expectations represent a major factor in enhancing treatment efficacy [22]. On the other hand, Hróbjartsson *et al*. [23] recently reported in a systematic review that there was no general evidence that a placebo had strong clinical effects other than on pain symptoms.

Another systematic review of randomised controlled trials of inhaled bronchodilators in patients with COPD suggested that a Hawthorne effect may have influenced SGRQ total domain scores in COPD trials [24]. Previous studies have indicated that the SGRQ total domain score decreases by 3 to 8.9 points under placebo treatment [25, 26]. In addition, in previous studies using placebo acupuncture for COPD, the difference in SGRQ total domain score between baseline and end of treatment was changed by an average of −2.3 to 0.5 in the placebo AG [12].

In our study, the SGRQ total domain score difference in the AG was −22.8 at week 52 compared with baseline. This major improvement could not be explained by the placebo effect alone. Further, we found no differences in expectations for acupuncture, which were measured before allocation. The majority of patients answered “Indifferent”, suggesting that the placebo effect likely had little to no impact on the results. In addition, the MCIDs for the SGRQ total domain score reported by Jones *et al*. [21] and Harma *et al*. [27] were −4 and −7, respectively.

However, although this study suggests that acupuncture treatment has an effect on the SGRQ total domain score, the results of this study should be interpreted with caution as the possibility of the Hawthorne effect or placebo effect occurring in the patients cannot be ruled out.

Regarding external validity, since this study examined the effect of adding acupuncture treatment to pharmacological therapy to the participating patients who have been receiving standard pharmacological therapy, and since the acupuncture treatment is a common practice, it is thought to be easy to implement in daily clinical practice.

### Acupuncture and QOL

A previous study reported that lower QOL in COPD patients is closely associated with DOE, decreased physical activity and frequent exacerbations [28].

In the current study, the AG showed improvement in shortness of breath (mMRC) in daily life and dyspnoea after exercise (MBS), and also showed a significant increase in 6MWD. These findings indicated that acupuncture could improve dyspnoea and exercise intolerance, resulting in improved QOL. In addition, the number of more than moderate exacerbations was significantly lower in the AG than in the UG in this study, which suggests a possibility that acupuncture decreases exacerbations in COPD patients. Therefore, the avoidance of exacerbations in COPD patients is indirectly related to the maintenance of QOL.

Furthermore, it has been reported that malnutrition in COPD affects QOL. In a study of outpatients with COPD, the poorer the nutritional status, the higher the total SGRQ score and by giving nutritional therapy to those patients, the total SGRQ score was improved by −3.61 units [29]. In the present study, since improvements of body mass index and nutritional biomarkers were observed in the AG compared with the UG, the improvement of nutritional status by acupuncture may also contribute to QOL.

We therefore that the QOL of the COPD patients in the present study was improved over a year by adding acupuncture to the usual treatment.

### Possible mechanisms underlying effects of acupuncture on COPD

In the present study of COPD patients, significant improvements in Dyspnoea MBS after 6MWT as well as mMRC were seen. The difference in Dyspnoea MBS between AG and UG was −3.0 at week 12 and −3.8 at week 52. Since the MCID for Dyspnoea MBS after 6MWT in COPD patients has been reported as −2 [30], acupuncture could have kept reducing DOE in COPD patients for 1 year.

Dyspnoea in COPD patients has multiple causes, with fatigue of the respiratory muscles being a significant contributing factor [31, 32]. When Kawakita *et al*. [33] created an experimental model of muscle fatigue by subjecting muscles to high loads, they found high electromyographic activity consistent with locomotor insufficiency. However, they reported that applying acupuncture to fatigued muscles not only ameliorated feelings of fatigue, but also suppressed electromyographic activity, enabling resumption of exercise. As many of the acupuncture points used in this study correspond to accessory respiratory muscles and muscles used for walking, similar phenomena may have been evoked. Further, significant increases in forced vital capacity, FEV_1_, carbon monoxide diffusing capacity and respiratory muscle strength such as maximal expiratory pressure and maximal inspiratory pressure, and decreases in functional residual capacity, residual volume and total lung capacity seen in the AG could have contributed to reductions in DOE.

COPD is a systemic inflammatory disease with increased blood levels of inflammatory mediators even in the stable phase [34]. The AG exhibited significant reductions in TNF-α, IL-6 and hs-CRP compared with the UG in this study. Recent studies have shown that acupuncture at ST36 (Zusanli) promotes the transmission of stimuli descending from the dorsal nucleus of the vagus nerve in the medulla to the adrenal medulla and the release of dopamine from the adrenal medulla, resulting in decreased levels of TNF-α, IL-1β and IL-6, all of which are elevated during systemic inflammation [35, 36]. Our results suggest that similar mechanisms may contributed to the observed decreases in levels of inflammatory mediators.

In this study, the AG showed significant improvements in body composition and haematological values at week 52 compared with the UG. Factors associated with malnutrition in COPD include elevated catabolism, caused by decreased ventilatory efficiency due to obstructive ventilatory disorder and increased respiratory muscle workload due to respiratory muscle fatigue [37]. Since acupuncture treatment improved respiratory function and ameliorated muscle fatigue in this study, improvements in nutritional status might have been achieved *via* improved energy efficiency related to respiration.

### Study limitations

Some limitations to this study need to be kept in mind when interpreting the results. First, all patients were already receiving standard pharmacotherapies for COPD because recruitment of patients not receiving medication was not possible. Changes in outcome measures in the present trial thus may have been achieved not only by acupuncture, but also by synergic effects with pharmacotherapies. Second, since placebo acupuncture was not used in this study, the possibility of a Hawthorne effect or placebo effect occurring in patients in the AG as a result of participating in the study cannot be denied. Therefore, the results of this study must be interpreted with caution. Third, in this study, the AG had more visits to hospitals or clinics than the control group, which may have potentially influenced physical activity. Fourth, the present study only included patients who were able to attend an outpatient clinic for acupuncture treatment once a week for 1 year, and a larger sample size would be needed for generalisation.

### Conclusion

Our results indicate that QOL and dyspnoea were dramatically improved in AG patients without major adverse reactions, suggesting that acupuncture may represent a useful modality for managing stable COPD. Acupuncture shows potential for having overall effects of improving anti-inflammatory responses, nutritional status, respiratory function and muscle strength.

## Supplementary material

10.1183/23120541.00668-2024.Supp1 **Please note:** supplementary material is not edited by the Editorial Office, and is uploaded as it has been supplied by the author.
Supplementary material 00668-2024.SUPPLEMENT
Statistical analysis plan 00668-2024.SUPPLEMENT2
Supplementary tables 00668-2024.SUPPLEMENT3
