# Supplementary material for: Effectiveness of a long-term acupuncture treatment in patients with COPD: a randomised controlled trial
Source: ERJ Open Res. 2025 May 19;11(3):00668-2024. doi: 10.1183/23120541.00668-2024 (PMC12086826; doi:10.1183/23120541.00668-2024)

Effect of long term intervention with acupuncture on COPD: A randomized controlled trial.　Long term COPD Acupuncture Trial (LCAT)

Clinical sites:

1. The Tazuke Kofukai Medical Research Institute Kitano Hospital Respiratory Medicine and 12thDivision
2. Toneyama National Hospital Respiratory Medicine
3. Ikeoka Clinic
4. Minato Clinic

Data Management and Statistical Centers:

Kyoto University School of Public Health, Dept. of Health Informatics and The Tazuke Kofukai Medical Research Institute, Dept. of clinical data center.

Trial registration

The protocol has been registered in University hospital Medical Information Network Clinical Trials Registry (UMIN-CTR), with approval number UMIN000008934.

Protocol version

Version number: 1.0. Version data: July 17th, 2012

Version number: 2.0. Version data: September 20th, 2013

Version number: 3.0. Version data: June 14th, 2017(Final version)

Funding

This study is carried out as a project of the Tazuke-Kofukai medical research institute (No:H-252013), the Japan society of acupuncture and moxibustion (JSAM) (No: jsam201201) research grant, and Japan Agency for Medical Research and Development (AMED) Research Project for Improving Quality in Healthcare and Collecting Scientific Evidence on Integrative Medicine (No: 18lk0310045h0001).

Roles and responsibilities

The protocol was designed by Suzuki M, Takahashi Y, Maekura T, Miki K and Fukui M. The funder had no role in study design, collection, management, analysis, and interpretation of data; writing of the report; and the decision to submit the report for publication.

Table of Contents

1. Title of the research 4
2. Site-specific information for the research 4
3. Trial registration 5
4. Roles and responsibilities 5
5. Objectives and significance of the research 5
6. Method and time period of the research 6
   - Study design 6
   - Blinding 7
   - Protocol of the Study 7
   - Random allocation 9
   - Method of acupuncture treatment 9
   - Outcome Measures 10
   - Statistical Analysis 13
   - Sample size 15
   - Period of Clinical Trial 15
7. Enrolling Criteria of research subjects 16
8. Basis of scientific validity for implementing the research 16
9. Procedures for obtaining informed consent 18
10. Handling of personal information 19
11. Burdens to be caused on the research subjects and predicted risks and benefits, including comprehensive assessment of such burdens, risks and benefits as well as measures to minimize those burdens and risks 19
12. Means for storage and disposal of information 20
13. Matters to be reported to the chief executive of the research implementing entity and procedures for such reports 21
14. Status of research-related conflicts of interest of the research implementing entity, such as research fund resources, as well as research-related conflicts of interest of such investigator, etc., such as his/her individual income 21
15. Means to disclose information on research 21
16. Means to respond to the consultation, etc. made by the research subjects, etc. and other individuals concerned 21
17. When the research involves any financial expenditure on or remuneration for the research subject, etc., a statement to the effect that and details of such 22
18. When the research involves invasiveness, means to respond in cases of serious adverse event 22

19. When the research involves any invasiveness, whether or not compensation will be offered for research-related injury and detail of such compensation 22
20. When the research involves any medical technique beyond usual medical practice, response related to the healthcare delivery to the research subjects after the research
23
21. When monitoring or audit is performed, organizational framework and procedures for such 23
References 24
Reference Materials
Material A-1, Procedure of Clinical Study 26
Material A-2, Study design schedule 27
Material B, GOLD Classification 28
Material C-1, Method of Acupuncture 28
Material C-2, Method of Acupuncture(table) 30
Material D, Modified Borg Scale 31
Material E, Modified MRC Dyspnea Scale 31
Material F, Evaluation of Acute Aggravation 32
Material G, Organization Structure 32

1. Title of the research

Effect of long term intervention with acupuncture on COPD: A randomized controlled trial.
Long term COPD Acupuncture Trial (LCAT)

2. Site-specific information for the research

Main researcher

- The Tazuke Kofukai Medical Research Institute Kitano Hospital Respiratory Center 12th Division.
  Director, Motonari Fukui
  〒530-8480　2-4-20 Ohgimachi, Kita-ku, Osaka Tel：06-6312-1221

Main Clinical Researcher

- The Tazuke Kofukai Medical Research Institute Kitano Hospital, 12th Division
  Chief, Masao Suzuki
  〒530-8480　2-4-20 Ohgimachi, Kita-ku, Osaka Tel：06-6312-1221

Researchers in charge

- The Tazuke Kofukai Medical Research Institute Kitano Hospital Respiratory Center
  Director, Satoshi Marumo. Vice Director, Ryo Itotani. Vice Director, Yuko Katayama.
  Physician, Daiki Inoue. Physician, Takamasa Kitajima. Physician, Hiroshi Shima.
  Physician, Masahiro Hirata.
  〒530-8480 2-4-20 Ohgimachi, Kita-ku, Osaka Tel：06-6312-1221

Collaboration facilities and collaborator

- Ikeoka Clinic. Director, Kiyomitsu Ikeoka
  〒536-0008 1-18-13 Sekime Joto-ku, Osaka Tel：06-6931-6665
- Minato Clinic. Director, Hiroshi Minato.
  〒531-0063 1-4 Nagaehigashi Kita-ku, Osaka Tel: 06-6356-7381
- Department of Clinical Research Toneyama National Hospital.
  Principal researcher and General Manager, Keisuke Miki.
  〒560-0045 5-1-1 Toneyama, Toyonaka, Osaka Tel：06-6853-2001
- Aizu Medical center of Fukusima Medical University. Associate professor, Masao Suzuki.
  〒969-3492 21-2 Kawahigashi, Aizuwakamatsu city Fukushima.

Researchers in charge

- Toneyama National Hospital Respiratory Medicine
  Director, Seigo Kitada. Head physician, Mari Miki. Head physician Kenji Yoshimura.
  Physician, Kazuyuki Tsujino. physician, Hiroyuki Kagawa. physician, Yohei Oshitani.

physician, Kouhei Nishida. Physician, Yuki Hosono. physician, Yoshiko Kureya. physician, Tatsuya Edahiro. physician, Hiroyuki Kurebe. physician, Kenya Fujikawa. physician, Naoko Hashimoto.

〒560-0045 5-1-1 Toneyama, Toyonaka, Osaka Tel：06-6853-2001

Statistician

・ Kyoto University School of Public Health, Dept. of Health Informatics
  Associate Professor, Yoshimitsu Takahashi
  〒606-8501 Yoshida Konoe, Sakyo-ku, Kyoto Tel：075-753-9477

Persons in charge of acupuncture treatment

・ The Tazuke Kofukai Medical Research Institute Kitano Hospital, 12th Division
  Chief, Masao Suzuki and Researcher, Shingo Saitho
  〒530-8480 2-4-20 Ohgimachi, Kita-ku, Osaka Tel：06-6312-1221
・ Ikeoka Acupuncture and Moxibustion Clinic
  Director, Miki Konishi
  〒536-0008 1-18-13 Sekime Joto-ku, Osaka Tel：06-6930-5606
・ Toneyama National Hospital Respiratory Medicine
  Hospital Researcher, Tomonori Maekura
  〒560-0045 5-1-1 Toneyama, Toyonaka, Osaka Tel：06-6853-2001

Main researcher and Main Clinical researcher: manages the study design, the entire testing, and decides the cancellation of the study.

Physician in charge: performs patients' management and informed consent.

Person in charge of acupuncture treatment: is an acupuncturist who has the certification of national qualification.

Statistician: performs statistical analysis on evaluation items and relevant data of this study.

3. Trial registration

The protocol has been registered in University hospital Medical Information Network Clinical Trials Registry (UMIN-CTR), with approval number UMIN000008934.

4. Roles and responsibilities

The protocol was designed by Suzuki M, Takahashi Y, and Fukui M. The funder had no role in study design, collection, management, analysis, and interpretation of data; writing of the report; and the decision to submit the report for publication.

5. Objectives and significance of the research

● Objectives of the research

Purpose of our clinical trial is to study effects of acupuncture treatment on COPD patients for one year and evaluate its clinical effects of alleviating dyspnea on exertion and improving QOL (Quality of Life) of the patients.

● Significance of the research

Chronic Obstructive Pulmonary Disease (COPD) impairs patients' activities in daily life, their quality of life (QOL) and social activities due to dyspnea on exertion. Its prevalence is estimated to reach 5.3 million in Japan[1)].

Main treatments for COPD currently are pharmacotherapy, oxygen therapy, respiratory rehabilitation, and nutritional counselling, but these treatments are not for curing the disease but for controlling the symptoms. And although it is reported important to ameliorate Dyspnea on Exertion (DOE) and improve QOL in order to manage COPD for a long period of time[2)], it cannot be easily done by the current limited methods. Therefore, we consider it is necessary to supplement the current treatment methods.

There are some clinical studies reported which used acupuncture for COPD. Our clinical study using acupuncture for patients with COPD, where we gave acupuncture treatment once a week for 10 weeks for 30 cases with matched pair control, found that the mean Borg scale score, which indicates degree of dyspnea on exertion after 6-minutes' walk, was 6.43 (SD 3.46) for control group, and 2.17 (SD 2.66) for acupuncture treatment group[3)]. Since this preceding study indicated a certain effectiveness of acupuncture treatment for DOE of COPD, we then implemented a randomized control trial (RCT) with 12 weeks intervention and found that the mean Borg scale score after 6-minutes' walk were 4.6 (SD 2.8) for placebo group (n=32) and 1.9 (SD 1.5) for acupuncture treatment group, i.e., a significant improvement in acupuncture treatment group was seen. This study also found that acupuncture treatment group showed significant improvements in QOL of COPD patients (St George's Respiratory Questionnaire) and in nutritional evaluation (Body mass index, Prealbumin)[4)]. Also, regarding the safety of acupuncture treatment, previous studies have not found any harmful incidents which cause physical pain including pneumothorax or danger of life.

Although its safeness and short-term effect for COPD were found by these studies, in order to have acupuncture treatment set as one of the COPD treatment programs, we consider that it is necessary to evaluate its effects for the much longer time period. Since there has been no study considering long-term effects of acupuncture treatment on COPD, we plan to study effects of acupuncture treatment on COPD patients for one year and evaluate its clinical effects including QOL.

6. Method and time period of the research

- **Study Design**

This study is a multi-center study and will be conducted at 4 facilities in Osaka Prefecture. This study is planned according to the CONSORT (Consolidated Standards of Reporting. Trials) statement[5)], SPIRIT (Standard Protocol Items: Recommendations for Interventional Trials) guideline[6)], and STRICTA (STandards for Reporting Interventions in Clinical Trials of Acupuncture) guideline[7)].

Randomized parallel-group comparison study

Acupuncture Group (AG): Usual care and acupuncture treatment

Usual care Group (UG): Usual care only

- **Blinding**

The nature of the study does not allow patients and acupuncturists to be blinded. However, in this study, blinding will be performed on the doctors in charge, the data collectors (including the evaluator), the data inputters, and the statistical analysts. In addition, data collectors and data inputters will be implemented by independent persons who are not involved in this research.

At all facilities, acupuncture is conducted in completely private rooms, and measures are taken to prevent participants from meeting each other. Additionally, the acupuncture rooms are located in a separate building from the hospital to avoid contact between participants and medical personnel.

Study Schedule

Patients' entry period: From 15 January 2013 to 31 October 2018.

Patients' entry method

Recruitment of relevant patients by the physician in charge of outpatient treatment.

Period of intervention

Period of intervention for this study is one year (52 weeks) in maximum.

* Principal investigator is instructing the attending physician not to change the medication during the study in both groups. If a patient's condition worsened and treatment required a drug change, the drug change is temporarily permitted; however, when the patient's condition became stable, the previous treatment is resumed. The clinical researcher writes down about the medication and other treatment conditions on the specified record cards, which are preserved.

- **Protocol of the Study (Material A-1, A-2)**

Informed consent and entry evaluation (various tests) of the patients are obtained at the

master hospital (Kitano Hospital Respiratory Center) as below.

A. The physician in charge evaluates whether a COPD patient fulfills the selection criteria. (Baseline, -4 to -2 Weeks)

B. The physician in charge explains this clinical trial to the patient and confirms the patient's intention orally whether he/she is "willing" or "not willing" to participate in this clinical trial. When the patient is willing to participate, the physician in charge notifies the clinical researcher of the patient's willingness, and then the clinical researcher sets up a meeting with the patient at the master hospital to obtain informed consent on this clinical trial from the patient. If the patient shows again the intention of participation at the meeting, informed consent of the patient is confirmed by a letter of consent. (Baseline, -2 week)

(At this stage, it is explained that even if the patient wants to have acupuncture treatment, he/she may not be able to have it.)

C. For the patient whose letter of consent is completed, the physician in charge at the master hospital orders the following tests as the initial evaluation. (Baseline, 0 Week)

In that case, if the patient visits the master hospital for the first time, the patient has to be registered as the first visit patient and to have the examination by the physician in charge and various tests according to the instruction given by the physician in charge at the master hospital. The reason to have the patient registered is to be able to take action immediately to avoid risk in case of sudden change during examinations such as the 6 minutes-walk or the detailed respiratory function test.

1. Respiratory function test: A detailed respiratory function test, B respiratory resistance (Impulse Oscillation System)
2. Blood test

Note: In order to alleviate the burden on a patient, if the patient's condition is stable, data for the tests 1 and 2 obtained within 1 months can be used. SGRQ questionnaire

4. Exercise tolerance test (6-minute walk test such as, dyspnea on exertion [modified Borg Scale], walking distance, lowest SpO2, highest pulse and lower limb fatigue [modified Borg Scale])
5. Test on respiratory muscle strength
6. Physical evaluation test (height, weight, upper arm circumference, triceps brachii muscle skinfold thickness, grasping power, thorax excursion)

* <u>The evaluators are conducted by inspectors who are not related to this study.</u>

D. When the initial evaluation is completed, the clinical researcher registers the patient to the clinical trial and asks the allocation center (Kyoto university) to allocate the patient.

(Baseline 0 week)

After completing the allocation process, the clinical researcher informs the participants which group they have been assigned to.

E. COPD patients who are allocated to AG (group with intervention) visit once a week one of the acupuncture treatment facilities collaborating with this study [The Tazuke Kofukai Medical Research Institute, Research Division for Integrating Oriental and Western Medicine (Osaka city), Ikeoka Acupuncture and Moxibustion Clinic (Osaka city), or Toneyama National Hospital Respiratory Medicine, Acupuncture and Moxibustion Room (Toyonaka city)] for 48 times (one year (52 weeks)). For each treatment, the person in charge of acupuncture treatment describes the progress and medical regimen on the acupuncture treatment record cards. The regular periodical examination for COPD patients of both AG and UG is given on the date designated by the physician in charge.

F. Tests shown above for initial evaluation are also to be given or ordered after 12 weeks and one year (52 weeks).

- **Random allocation**

The allocation of the participants to study groups is carried out in a centralized manner by setting up the allocation center at Kyoto University. After the baseline assessment and stratification by the GOLD classification (II or III or IV)(Material B), an independent investigator randomly assigned eligible patients to the usual care group(UG) or the acupuncture group(AG) in a 1:1 ratio using a computer-generated random allocation sequence(Microsoft excel. Microsoft Co., Washington D.C.,U.S.A) with permutated block design and a block size of four.

To maintain blinding in physician, outcome assessors, and statisticians, the allocation sequence was concealed until the end of the study.

Statistical analysis is done by the statistician under masking. The allocation is labeled as “A” or “B”, and after the allocation is notified, the acupuncturist will set either “A” as usual care and “B” as acupuncture or the other way around. The clinical researcher and acupuncturists shall not reveal the statistician in which group a patient is allocated from the onset of the trial until the final statistical analysis is reported. The blind to the attending physician should not tell the result of the assignment unless an adverse event occurs. Also, the patient must not inform the physician in charge of the allocation result.

- **Method of acupuncture treatment (Material C1, C2)**

Acupuncture treatments are performed once a week for 48 times during the one year (52 weeks). Method of acupuncture treatment utilizes the acupuncture points which are reported effective for COPD by prior researches[3-4)].

Acupuncture needles and positions of acupuncture points to be used.

Acupuncture needles used are disposable needles (Seirin Co., Ltd), 30-40mm in length and 0.14-0.25mm in thickness. The WHO standardized acupuncture points used in this study are as follows: ① LU1 (Zhongfu), ② LU9 (Taiyuan), ③ CV4 (Guanyuan), ④ CV12 (Zhongwan), ⑤ CV17 (Zhanzhong), ⑥ LI18 (Futu), ⑦ KI3 (Taixi), ⑧ ST36 (Zusanli), ⑨ BL10 (Tianzhu), ⑩ BL13 (Feishu), ⑪ BL20 (Pishu), ⑫ BL23 (Shenshu)[8)]. (See figures and tables at the end of this document)

After inserting an acupuncture needle into both sides acupuncture point and the patient feels the deqi, the needle is rotated in a direction for 3 minutes to keep the deqi.

This procedure is performed on all acupuncture points. Total treatment time is about 60 minutes.

Acupuncturists who participate in this study are required to hold a national license and possess a minimum of 10 years of clinical experience. In addition, they must attend a five-week intensive program on acupuncture as part of the study.

・Acupuncturists participating in the study must meet the acceptance criteria within five weeks of receiving weekly evaluations from their instructors.

The evaluation consists of five items, with five criteria.

These include: Q1 Correct location of acupuncture point, Q2 Appropriate order of needle insertion, Q3 Appropriate needle insertion technique, Q4 Correct needle rotation technique, and Q5 Appropriate deqi level.

・The evaluation criteria require a minimum total score of 20 points to pass. The criteria are rated on a scale of 1 to 5, where 1 means 'not at all appropriate,' 2 means 'slightly appropriate,' 3 means 'fairly appropriate,' 4 means 'very appropriate,' and 5 means 'extremely appropriate'.

To account for variations in technique among acupuncturists, practitioners will be assigned on a monthly rotation basis to patients in this study.

The drugs used for patients in both groups will remain unchanged throughout the study, except in cases where therapeutic drug changes are necessary due to exacerbation. However, patients will be instructed to resume the original drug as soon as their condition stabilizes.

- **Outcome Measures**

A. Primary outcome measure

QOL questionnaire: SGRQ (St. George’s Respiratory Questionnaire)[9)] Total domain in Japanese version.

*Main evaluate the difference one year (52 weeks) after the baseline.

*The SGRQ assessment is obtained by patients’ filling in the paper-format questionnaires in a hospital room where no research-related personnel around, and the questionnaires are

collected by hospital staff unrelated to the study.

B. Secondary outcome measures

As for Secondary and other outcome measures, the following outcome were evaluated at baseline, 12 weeks, and one year (52 weeks) later.

a: QOL questionnaire: SGRQ total domain difference 12 weeks after the baseline.

b: QOL questionnaire: SGRQ domain other than Total domain (Symptom, Activity, Impact).

c: The 6-minute walk test (6MWT) assesses dyspnea on exertion using the modified Borg Scale (Material D), walking distance, lowest oxygen saturation level, highest pulse count, and lower limb fatigue measured with the modified Borg Scale.

*Evaluate the difference 12 weeks and one year (52 weeks) after the baseline.

C. Other outcome measures

a. Nutritional measures: ① BMI (Body Mass Index), ② serum prealbumin, ③ serum albumin, ④ hemoglobin, ⑤ Midupper Arm Circumference (MAC), ⑥ Triceps Skinfold thickness (TSF), ⑦Arm Muscle Circumference (AMC), ⑧Arm Muscle Area (AMA), ⑨grasping power.

* Skinfold thickness measures to assess changes in fat mass using Harpenden skinfold calipers (Holtain) according to its standard methodology[10]. Measurement is performed on the non-dominant hand, and in the case of paralysis, on the non-paralyzed side. We evaluate the average value of the three measurements. The measurement of TSF performs as follows. Subjects are in a lateral decubitus position, the upper arm was flexed by 90 ° at the elbow joint, a mark was placed at the midpoint between the shoulder bladder projection and the ulnar olecranon projection, the skin 1 cm away from the mark was picked up to so that the fat layer is separated from the muscle, and we measure the thickness by sandwiching the marked part with a caliper. As for the measurement of SSF, with the patients in the lateral decubitus position, the same method as TSF is used at the portion of the shoulder blade lower corner. For MAC, the surroundings of the part mark for TSF is measure with a measuring tape. AMC and are calculate by this formula: [AMC(cm)=MAC-3.14×TSF(cm)] and [AMA(cm)=(MAC-3.14×TSF)2/4π], respectively.

b: MMRC (modified Medical Research Council) dyspnea scale (Material E)

c: Frequency and severity of acute aggravation of COPD are evaluated by dedicated daily records. The diary will be self-filled and will be submitted to an independent evaluator after the study period.

Contents of daily records * Definition and Evaluation of acute aggravation: Severity scale[11] (Material F)

Type 1 (sever): 3 major items are found

Type 2 (moderate): 2 major items are found

Type 3 (mild): 1major with any minor items are found

Major Items:

(1) Obvious worsening of dyspnea, (2) Obvious increase of sputum purulence,
(3) Obvious increase of sputum quantity

Minor Items:

(1) Upper respiratory tract infection lasting more than 5 days, (2) Fever without other obvious cause, (3) Increased wheezing,
(4) Increased cough, (5) Respiratory rate or heart rate increased more than 20%.

In addition, we evaluate the number of unscheduled outpatient visits and hospitalizations associated with exacerbation of COPD.

* Evaluation of Severity scale is recorded on the specified evaluation table (daily record).

d. To evaluate QOL, CAT(COPD Assessment Test)[12)] Japanese version is used. The CAT, a disease-specific health status questionnaire, provides a measure of disease severity from the patient's perspective. It contains 8 questions (cough, phlegm, chest tightness, breathlessness, activities, confidence, sleep, and energy) that pertain to a patient's COPD symptom burden. Each question consists of a 6-point differential scale, and the final score is between 0 and 40.

e. BODE index[13)] is calculated. Prognosis outcome measurement is the BODE index, which is a multidimensional index that includes four factors that predict the risk of death: BMI (B); degree of air flow obstruction (O); functional dyspnea (D); and exercise capacity (E), assessed by the 6MWD.

f. Thorax excursion; Evaluation of thorax mobility was made by measuring the difference of the girth of the chest between the RV level and TLC level on the basis of the fourth intercostal using a tape measure.

g. Precision Respiratory function test: lung volume and its subdivision, flow volume curve, residual volume, pulmonary diffusion capacity[14)].

h. Respiratory resistance: Impulse Oscillation System (IOS) is used to evaluate the respiratory resistance.

* IOS parameters are collect before conventional spirometry parameters using the MasterLab IOS System (Erich Jaeger, Würzburg, Germany). Calibration is performed using a single volume of air (3 L) at different flow rates and a reference resistance device (0.2 kPa/L/s). The patients wear a nose clip and a manufacturer-provided, oval, hard plastic mouthpiece to prevent the expired air from escaping. They are also requesting to

support their cheeks with their hands to decrease shunt compliance. Artifacts caused by coughing, breath-holding, swallowing, and vocalization are not included. A single, experienced, respiratory technician made all the IOS measurements. The parameters evaluated are resonant frequency (Fres), resistance at 5 Hz (R5), resistance at 20 Hz (R20), the difference between the resistance at 5 and 20 Hz (R5–R20), reactance at 5 Hz (X5), area of reactance (AX), and impedance at 5 Hz (Z5) as calculating by √X52+R52.

i. Test on respiratory muscle strength: Maximum respiratory muscle strength at expiration and inhalation is evaluated by maximum mouth pressure.

* The maximum inspiratory mouth pressure (MIP) and maximum expiratory mouth pressure (MEP) were measured using a standard mouthpiece and a device (Vitaropower KH115, Chest MI Co. Ltd., Tokyo, Japan) according to American Thoracic Society/European Respiratory Society[15)].

j. Inflammatory biomarkers: Interleukin-6 (IL-6), Tumor Necrosis Factor-α(TNF-α), and High sensitivity CRP are measured.

The reason of the measurement: In recent years, it has been reported that COPD is a chronic systemic inflammatory disease and that systematic inflammation leads to worsening of COPD and causes comorbidities. On the other hand, it has been reported that acupuncture has an anti-inflammatory effect, and our previous study found some improvements in inflammation biomarkers related to COPD.

*Evaluate the difference 12 weeks and one year (52 weeks) after the baseline.

k. Patients' expectations for acupuncture

Expectations for acupuncture were evaluated on the following Six-level Likert scale. Evaluation is performed at the time of baseline evaluation after informed consent (conducted before allocation).

"0: I have no expectations, 1: I do not expect much, 2: Indifferent, 3: I'm expecting a little, 4: Have medium expectations, 5: I have high expectations"

*Patients' expectations for acupuncture are assessed at baseline only.

*All evaluations are carried out by independent evaluators not involved in the research.

· **Statistical Analysis**

1. Handling of cases and target group for analysis

In this study, cases enrolled in clinical trials and excluding dropouts will be the main analysis group.

2. Method of analysis

In this study, based on data analysis is performed by setting two analysis groups, FAS (Full Analyze Set) and PPS (Per Protocol Set).

FAS and PPS are defined as follows.

FAS: All assigned patients except for dropped out patients

PPS: Patients who achieved 40 or more acupuncture treatments in one year, excluding dropout patients.

Distributions of all the outcome measures at baseline are shown.

For the primary outcome measures, the secondary outcome measures, and other outcome measures, ratios or means of the differences between baseline and 12 weeks and between baseline and 52 weeks are calculated.

For all outcome measures, we look at the changes of variables in 12 weeks (from baseline to 12 weeks later) and in 52 weeks (from baseline to 52 weeks later). And then, for main outcome measure and secondary outcome measures, we compare these 12-week-change and 52-week-change using the analysis of covariance (ANCOVA) with baseline values and GOLD classification as covariates and treatment group as the factor of interest.

For the change from baseline in other outcome measures, an independent sample t-test is use.

Continuous variables and ordinal variables are shown as the means (standard deviation), and their significant differences are shown by 95% confidence intervals (CI) and p-values (significance level 0.05). Count variables are shown as the number of counts and percentages, and X2 test or Fisher's exact test results are shown with p-values (significance level 0.05).

For the changes in arterial blood oxygen saturation and in pulse during the 6-minute walk, we use the mixed effects model to analyze the difference between the 12-week-change and 52-week change. In this mixed effects model, the fixed effects are groups and times, the interaction terms are group x time, the intercept of random effects is individual, the slope is time, and the estimation method is the maximum likelihood method.

In this study, we perform the analysis without imputing missing values in the main analysis. In addition, as a post hoc sensitivity analysis, analysis of covariance will be performed with multiple imputation for the primary and secondary outcome measures, including missing data for dropouts. For dropout cases, multiple imputation is performed assuming MAR (Missing At Random), and when doing so, variables that exhibit a correlation (>0.5) with the baseline values of each evaluation item and the missing variables are used as auxiliary variables.

All main analyses are carried out with STATA-16 (StataCorp LLC, College Station, TX, USA).

All data are entered by an independent data entry personnel not involved in the study.

A combination of machine-generated alphabet letters was assigned to each patient and then the data were randomized so that the individual patient cannot be identified in the data without the collation list. Furthermore, no patient information will be revealed to all

those involved in the study until the statistical analysis is complete.
The statistician must perform the analysis with the grouping and patient information masked.
3.Preparation of the statistical analysis report.
After all of the case-handling is done and data are fixed, statistical analyses are performed and the statistical analysis report is prepared.

- **Sample Size**

1. The target number of cases is 44.
2. The sample size was found using the test of difference (two-sided) in the mean value of the difference of SGRQ (Total domain) values between before and after 52weeks of the acupuncture treatment from our preceding research results. Considering the results of our preceding research and the report on Minimal clinically importance (MCID) of SGRQ (total domain) as 4 units16), setting the absolute value of the difference to be detected as 10 units, standard deviation(SD)10.0, the significance level as 0.05, and power as 0.8, the sample sizes per group was calculated to be 17 (two groups 34). This trial considers clinical effects for one year long, a certain number of dropouts is expected. Therefore, assuming approximately 30 percent (10 casess) may drop out, about 22 cases for each group is necessary (the final sample size was 44).

Table Sample size (two groups)

τ	Power (%)	Standard deviation			
		9	9.5	10.0	10.5
10	90	37	40	45	49
	80	28	31	**34**	37
	70	23	25	27	30
	60	18	20	22	24
	50	15	16	18	20

Criteria of Intervention Cancellation

Cases to be considered as intervention cancellation

1. when a patient requests to stop acupuncture treatment
2. when the physician in charge considers further acupuncture treatment is not appropriate (including the request from patient).
3. When a patient is hospitalized due to the acute aggravation of COPD or other reasons and intervention by acupuncture treatment becomes difficult, the physician in charge or the main researcher can cancel the intervention.
4. When an acupuncture treatment facility collaborating with this research cannot

continue acupuncture treatment, the main researcher can cancel the intervention.

- **Procedure of Trial Stoppage (cancellation)**

1. During the trial period, in case the physician in charge recognizes that the safety of acupuncture treatment cannot be secured, the main researcher shall be consulted and can decide to cancel the trial.
2. If the cancellation of the trial is decided, all who are involved in this trial should be notified within one week and the trial is cancelled.

Period of Clinical Trial (plans)

From July 17th, 2012 to December 31th, 2020

Date trial data considered complete：February 29th, 2020

Date analysis concluded: August 30th, 2020. (Additional Analysis; January 31th 2023)

7. Enrolling Criteria of research subjects.

Patients with COPD to be included in this study are those who are diagnosed with COPD according to Global Initiative for Chronic Obstructive Lung Disease (GOLD)[2)] by NHLBI/WHO workshop report, who are able to visit the hospital as outpatients and fulfill the following criteria.

- **Inclusion criteria**

1. Patient diagnosed as COPD and able to visit as outpatient.
2. Patient who had been judged as stable condition by the physician.
3. Those who were rated as grade 1 or more according to MRC criteria.
4. Those who were classified as stage II or more advanced to GOLD criteria.[2)](Material F)

"Stable" here means when a COPD patients were clinically stable with no history of infections or exacerbation of respiratory symptoms and no changes in medication within the 3 months preceding the study outset, and had no signs of edema.

- **Exclusion criteria at the onset of the study**

1. Those who have heart failure (The cor pulmonale is excluded), collagen disease (RA and PM/DM, etc.), malignant tumor, or severe mental disorders.
2. The patient who were in concurrent respiratory rehabilitation program.

8. Basis of scientific validity for implementing the research.

Several overlapping causes lead to COPD patients' dyspnea on exertion: dynamic hyperinflation of the lung associated with peripheral airway obstruction may be generally considered the main cause, but the fatigue of respiratory muscle (including accessory muscle) is also a major cause of dyspnea on exertion[17)]. Not only that, brain function (central function) is also considered to affect dyspnea, and the stimulation of emotional

and behavioral centers such as amygdala, hypothalamus and cerebellum are recently reported to be involved[18-19].

On the other hand, the effects of acupuncture on COPD at the peripheral level are relief of muscle tone and improvement of muscle fatigue. It is known that where muscle fatigue is occurring, not only the algesic substance such as bradykinin is accumulated in muscles, but also abnormal excitation is found in the muscles. Acupuncture stimulation to such muscles can suppress the abnormal excitation of the muscles and metabolize the algesic substance by increasing the muscle blood flow. Acupuncture has effects of muscle tone relief and fatigue improvement by these mechanisms[20]. As for the central level effect, it is known that acupuncture given to the periphery can produce a strong analgesic effect by activating endogenous opioid (enkephalin, dynorphin, ßendorphin) from periaqueductal grey (PAG) through polymodal receptor via C-fiber[21]. Also, it has been confirmed by fMRI that acupuncture stimulation has the suppression effect on emotional and behavioral centers such as amygdala and cerebellum[22]. Although it has been known in normal clinical practice that opioids can relieve dyspnea in patients, opioids used as a drug are not usually applied to COPD patients since they may cause respiratory depression. Endogenous opioid activated by acupuncture stimulation, on the other hand, does not cause respiratory depression; therefore, it can be proposed as a safe treatment.

Furthermore, COPD has been reported recently to be not just a respiratory disease but a systemic and progressive inflammatory disease. Especially, it has been reported that with the increase of inflammatory cytokines such as IL-6, IL-8, TNF-α, and TGF-ß, inflammation spreads not merely lung but systemically and occurs with comorbidities such as hypertension, diabetes and various cancers[23]. Moreover, acupuncture stimulation has been reported recently such as in Nature Medicine to be effective in suppressing inflammatory cytokines such as IL-6, IL-8, TNF-α, and TGF-ß[24]. If inflammatory cytokines can be suppressed by acupuncture, acupuncture would be able to prevent not only the severity of COPD but also its comorbidities. Therefore, we consider that to perform research on acupuncture for COPD is highly significant and that the scientific rationality of this research has a sound basis.

As for the expected research results, the improvements of QOL and prognosis are possible. COPD causes deterioration of QoL due to dyspnea on exertion, the chief complaint, and COPD gets worse from disuse syndrome due to the decreased activity which then leads to the bedridden condition for a long period of time. Therefore, it is necessary to sustain and improve QoL of COPD patients by reducing and preventing dyspnea. COPD guideline does report that exacerbation of dyspnea and declining QoL are related to the patient’s prognosis. Despite the indication of the guideline, many COPD patients are still deprived of

their daily lives and social activities due to dyspnea on exertion. We need to expand and compensate the current COPD treatment. We consider that the expected results from our study would improve conditions of dyspnea on exertion and QoL and contribute to improving the prognosis of COPD patients by utilizing acupuncture. COPD is also a systemic inflammatory disease, it is also expected that acupuncture would reduce inflammation. Since our research aims to expand the existing medical practice, we consider that our research could significantly contribute to the academics.

9. Procedures for obtaining informed consent.

This study is performed based on "Declaration of Helsinki," "Ethical Guidelines for Epidemiological Studies," and "Regarding the Enforcement of the Ethical Guidelines for Clinical Research."[25-26] regarding the protection of the rights of subjects,

The document of information on the research and informed consent, which were approved by the ethical review committee of the master hospital, are given to the patient with the adequate amount of explanation in documentation and by word of mouth. Patient's consent by free will is obtained in the document. In the cases such as when information of danger which may influence patient's consent is found or when there is an alteration of the research plan, etc., which may influence patient's consent, the patients are to be informed promptly and be asked about their willingness to continue the participation in the research in advance, and after the alteration of the document of information on the research and informed consent are approved by the ethical review committee of each facilities, patient's consent is obtained again.

- The items below shall be included in the documents of explanation and informed consent.

(1) Title of the research and its approval given by the chief executive of the research institute.
(2) Names of the research institute and the principal investigator.
(3) Objective and significance of the research.
(4) Method and period of the research.
(5) Reasons why you are selected as a research subject.
(6) Burdens, risks, and benefits of the research subject.
(7) You can withdraw your consent at any time.
(8) You will not be disadvantaged if you do not agree to participate or if you withdraw your consent.
(9) Methods of information disclosure regarding the research.
(10) Study subject's request of acquiring and/or browsing the research materials is

allowed within the extent that does not violate the protection of personal information of other research subjects and that does not interfere with research activity, and its procedure is shown.

(11) Handling of personal information.

(12) Means for storage and disposal of samples/information.

(13) Research funds and conflict of interest.

(14) Responding to consultation requests from research subjects.

(15) Contents of the financial burden on or reward to the research subject, if it exists.

(16) If the research involves any medical activities beyond ordinary medical care, provision of medical care to the subject after conducting research.

(17) If the research involves any invasiveness, whether or not compensation will be offered for research-related injury and its contents.

(18) If there is a possibility of secondary use of samples/information and of providing it to other research institutes, its expected scenarios.

(19) In the case of intervention study involving invasiveness, the monitoring/auditing personnel and ethics committee will browse the samples/information within the necessary extent assuming the protection of personal information of the research subjects.

10. Handling of personal information.

Samples used in this research shall be anonymized to maintain the reliability of research analysis. Specifically, after creating the data using numbers attached to each individual of the obtained medical information, data is anonymized by deleting personal information. Both the anonymized data and the correspondence table of number and person are separately recorded and stored in external storage media. After the end of the research period, researchers keep the anonymized data and the principle (main) researcher keeps the correspondence table in locked storage cabinets at each research sites.

Also, the computers that store the anonymized data and the correspondence table are ones disconnected from other computers.

11. Burdens to be caused on the research subjects and predicted risks and benefits, including comprehensive assessment of such burdens, risks and benefits as well as measures to minimize those burdens and risks.

1. This study performs exercise stress test to study dyspnea on exertion for COPD patients, and it is possible that this stress test causes a patient to suffer strong dyspnea on exertion. Therefore, a physician specialized in pulmonology not involved in this study

attends the test and performs appropriate treatment such as oxygen inhalation if necessary.

2. If any side reactions (internal bleeding, muscle pain, fatigue, etc.) are caused by acupuncture treatment, we keep the patient at rest, and if there are no improvements, treatments are given by the physician in charge at each medical facility. Also, in order to minimize side reactions, we use as thin an acupuncture device as possible.
3. As a serious error, it is conceivable that pneumothorax is caused by acupuncture since alveoli of COPD patients are vulnerable. This risk can be avoided by limiting insertion up to 5mm at acupuncture points where pneumothorax might occur. If pneumothorax should occur, the physician in the respiratory department would immediately diagnose it and take appropriate measures.
4. If a test subject patient has any anxiety or seeks consultation during or after the test, the physician in charge or the clinical researcher are available for consultation.

12. Means for storage and disposal of information.

1) Methods for storage and disposal of information.

Information material utilized in our study

1. Information on paper medium

① QOL questionnaire (SGRQ, CAT), ② 6-minute walk test (record paper), ③ nutritional assessment (record paper), ④ diary, ⑤ detailed respiratory function test record paper, ⑥ respiratory resistance record paper, ⑦ respiratory muscle strength record paper, ⑧ blood test record paper, ⑨ MMRC dyspnea scale, ⑩ physical measurement record paper, ⑪ clinical records exclusive to study

Storage location: Medical research institute kitano hospital data management center.

Person in charge: Manager

Storage method: Papers are put in the cart for exclusive use, which is stored in the cabinet with lock.

Duration of storage: 10 years from the end of the study

Handling of data or method of destruction after the end of the study: Papers will be cut by cutting machine and discarded after 10 years of storage duration after the end of the study.

Method of anonymization: Data are anonymized as described in “8. Handling of personal information”

2. Dataset (hard disk for exclusive use)

Storage location: Medical research institute kitano hospital data management center.

Person in charge: Manager
Storage method: Hard disks are put in the cart for exclusive use, which is stored in the cabinet with lock.
Duration of storage: 10 years from the end of the study
Handling of data or method of destruction after the end of the study: Hard disks will be initialized and discarded after 10 years of storage duration after the end of the study.
Method of anonymization: Data are anonymized as described in “10. Handling of personal information”

13. Matters to be reported to the chief executive of the research implementing entity and procedures for such reports.

Research progress report will be submitted to the chief executive of the Kitano Hospital once a year until the research ends. Also, if there are any occurrences of serious adverse events or facts which impair ethical validity or scientific rationality during the research, which is considered to influence the continuation of the research, we will report to the chief executive of the Kitano Hospital according to the procedure of the ethical review committee of the Kitano Hospital

14. Status of research-related conflicts of interest of the research implementing entity, such as research fund resources, as well as research-related conflicts of interest of such investigator, etc., such as his/her individual income.

This study is carried out as a project of the Tazuke-Kofukai medical research institute research grant, the Japan Society of Acupuncture and Moxibustion (JSAM) research grant, and Japan Agency for Medical Research and Development (AMED) Research Project for Improving Quality in Healthcare and Collecting Scientific Evidence on Integrative Medicine. However, it is considered that the design of this study and results will not have any conflict of interest.

Each researcher, including the main researcher, physicians in charge, clinical researcher, and persons in charge of acupuncture treatment, submits self-declaration on conflict of interest to the committees that manage conflicts of interest of each facility and separately receives a review based on the conflict-of-interest guidelines.

15. Means to disclose information on research.

This study is carried out as a project of the Tazuke-Kofukai medical research institute research grant, the Japan Society of Acupuncture and Moxibustion(JSAM) research grant and Japan Agency for Medical Research and Development (AMED), information on research

will be disclosed as the project reports and medical papers. At that time, we also use general media such as newspapers through the internet and press clubs.

16. Means to respond to the consultation, etc. made by the research subjects, etc. and other individuals concerned.

The main researcher (Motonari Fukui), in principle, responds to the consultation from the research subjects and other individuals concerned, and the person in charge of acupuncture treatment (Masao Suzuki) responds to the technical consultation regarding acupuncture.

Contact: 06-6312-1221(main number of the Kitano Hospital)

17. When the research involves any financial expenditure on or remuneration for the research subject, a statement to the effect that and details of such.

The costs of tests performed in this clinical study, data of items listed below, which are the items to be used for normal outpatient service, are borne by patients. Therefore, the obtained data is used with patient’s permission. No other costs are borne by patients. There is no cost of acupuncture treatment.

○ Respiratory function test

○ Blood test (hemoglobin, albumin, prealbumin)

As for the remuneration for the research subject, when a subject cooperates with the participation in the research and has undergone the study period of one year, we will pay 20,000 yen as the compensation to the patient. The reason for the amount of 20,000 yen is based on examination fees (detailed respiratory function test and blood test) which are borne by the patient, consultation fee including first visit fee, and transportation expenses. The remuneration shall be covered by research expenses of the research grants.

18. When the research involves invasiveness, means to respond in cases of serious adverse event.

Pneumothorax is an adverse event that is of concern in this study. Although in our previous studies no pneumothorax has occurred with acupuncture for COPD patients, if an adverse event occurs, doctors in charge at each facility take appropriate action (such as bed rest by hospitalization, oxygen inhalation, insertion of trocar tube) promptly. If an adverse event occurs, the main researcher is notified immediately, and if it is decided that the pneumothorax occurred due to acupuncture, we will decide whether the study should be stopped.

19. When the research involves any invasiveness, whether or not compensation will be offered for research-related injury and detail of such compensation.

If an adverse event which is diagnosed by a physician and apparently caused by acupuncture treatment occurs, the treatment costs associated with the event will not be borne by the patient. The treatment costs for the aforementioned adverse event shall be borne by liability insurance.

20. When the research involves any medical technique beyond usual medical practice, response related to the healthcare delivery to the research subjects after the research.

Acupuncture treatment given in this study is not a part of the hospital treatment, but a generalized treatment given at acupuncture clinic. Therefore, when the study is over, and only if the patient wishes to continue acupuncture, we will introduce an acupuncture clinic in the area which the patient wishes.

21. When monitoring or audit is performed, organizational framework and procedures for such.

Monitoring

Monitoring is carried out with the aim of confirming whether this study is performed safely and in accordance with the research implementation plan and whether data is correctly collected. As a general rule, there is no inspection with facility visit. However, when there is a case that the main researcher deems necessary, inspection with facility visit will be conducted.

Implementation

The main researcher appoints Dr. Chifumi Iseki, a lecturer at the Department of Clinical Neuroscience, Yamagata University Graduate School of Medical Science, as a person who performs monitoring of the study considering she is appropriate for the task. The person who performs monitoring will prepare a report each time of monitoring and submit it to the main researcher. The main researcher will document the inspection and follow-up on monitoring reports.

Procedure

Items to be checked during the study: (1) confirmation of matters concerning consent; (2) confirmation of compliance with protocols and informed consent documents and on appropriateness of revision/use of the documents; (3) confirmation of storage status of the records; (4) collation of source material and report and confirmation of consistency; (5) confirmation of ethics committee procedure.

Items to be checked after the study: (1) confirmation of research procedure; (2) confirmation of document storage condition.

＜References＞

1. Fukuchi Y, Nishimura M, Ichinose M, et al : Prevalence of chronic obstructive pulmonary disease in japan: results from the Nippon COPD epidemiology (NICE) study. Eur Respir J 2001; 18 (suppl 33):275s.
2. Global Initiative for Chronic Obstructive Lung Disease. Global strategy for the diagnosis, management, and prevention of chronic obstructive pulmonary disease updated 2017. Available online at: http://goldcopd.org/gold-2017-global-strategy-diagnosis-management-prevention-copd/
3. Suzuki M, Namura K, Ohno Y, et al. The effect of acupuncture in the treatment of chronic obstructive pulmonary disease. J Altern Complement Med 2008; 14:1097-105.
4. Suzuki M, Muro S, Ando Y, et al. A Randomized, placebo-control trial of acupuncture in patients with chronic obstructive pulmonary disease(COPD): The COPD-Acupuncture Trial(CAT). Archi Intre Med. 2012; 11;172(11):878-86.
5. Schulz KF, Altman DG, Moher D; CONSORT Group. CONSORT 2010 statement: updated guidelines for reporting parallel group randomised trials. BMJ. 2010 23;340:c332. doi: 10.1136/bmj.c332.
6. An-Wen Chan, Jennifer M Tetzlaff, Douglas G Altman, Andreas Laupacis, et al. SPIRIT 2013 statement: defining standard protocol items for clinical trials. Ann Intern Med. 2013 5;158(3):200-7. doi: 10.7326/0003-4819-158-3-201302050-00583.
7. Hugh MacPherson 1, Douglas G Altman, Richard Hammerschlag, Li Youping, Wu Taixiang, Adrian White, David Moher, STRICTA Revision Group. Revised STandards for Reporting Interventions in Clinical Trials of Acupuncture (STRICTA): extending the CONSORT statement. PLoS Med. 2010 8;7(6):e1000261. doi: 10.1371/journal.pmed.1000261.
8. World Health Organization (WHO) Western Pacific Region. WHO Standard Acupuncture Point Locations in the Western Pacific Region. Manila: WHO Press Western Pacific; 2008.
9. Jones PW, Quirk FH, Baveystock CM, Littlejohns P. A selfcomplete measure of health

status for chronic airflow limitation. The St. George Respiratory Questionnaire. Am Rev Respir Dis. 1992; 145:1321-327.

10. Japanese Society of Nutritional Assessment. Japanese Anthropometric Reference Data: (JARD2001). Osaka, Japan: Medical Review Co; 2002.
11. Anthonisen NR, Manfreda J, Warren CP, Hershfield ES, Harding GK, Nelson NA. Antibiotic therapy in exacerbations of chronic obstructive pulmonary disease. Ann Intern Med. 1987 106(2):196-204.
12. P.W. Jones, G. Harding, P. Berry, et al. Development and first validation of the COPD Assessment Test. Eur Respir J 2009; 34: 648–654.
13. Celli BR, Cote CG, Marin JM, et al. The body-mass index, airflow obstruction, dyspnea, and exercise capacity index in chronic obstructive pulmonary disease. N Engl J Med 2004; 350:1005-12.
14. American Thoracic Society. Standardization of spirometry, 1994 update. Am J Respir Crit Care Med. 1995; 152: 1107-136.
15. ATS/ERS statement on respiratory muscle testing. Am J Respir Crit Care Med. 2002; 116: 518-624.
16. P.W. Jones. St. George's Respiratory Questionnaire: MCID. COPD. 2005 2(1):75-9.
17. Mahler DA, et.al. Recent advances in dyspnea. Chest. 2015 Jan;147(1):232-41.
18. Brannan S, et.al. Neuroimaging of cerebral activations and deactivations associated with hypercapnia and hunger for air. Proc Natl Acad Sci U S A. 2001 Feb 13;98(4):2029-34.
19. Evans KC, et.al. BOLD fMRI identifies limbic, paralimbic, and cerebellar activation during air hunger. J Neurophysiol. 2002 Sep;88(3):1500-11.
20. Kawakita K, et al.Experimental model of trigger points using eccentric exercise. J Musculoskeletal Pain. 2008;16:29-35.
21. Kawakita K, et.al. How do acupuncture and moxibustion act? - Focusing on the progress in Japanese acupuncture research -. J Pharmacol Sci. 2006;100(5):443-59.
22. Hui KK, et.al. The integrated response of the human cerebro-cerebellar and limbic systems to acupuncture stimulation at ST 36 as evidenced by fMRI. Neuroimage. 2005 Sep;27(3):479-96.
23. Gan WQ, et.al. Association between chronic obstructive pulmonary disease and systemic inflammation: a systematic review and a metaanalysis. Thorax 2004; 59: 574-80.
24. Torres-Rosas R, et.al. Dopamine mediates vagal modulation of the immune system by electroacupuncture. Nat Med. 2014; 20(3): 291-95.
25. Declaration of Helsinki （Translated by Japan Medical Association：

http://dl.med.or.jp/dl-med/wma/helsinki2013j.pdf）. in Japanese.
26. MEXT・MHLW：Ethical Guidelines for Medical and Health Research Involving Human Subjects. December 22, 2014..(https://www.mhlw.go.jp/file/06-Seisakujouhou-12600000-Seisakutoukatsukan/0000168764.pdf) in Japanese.

＜Reference Material＞

Material A-1 Procedure of Clinical Study

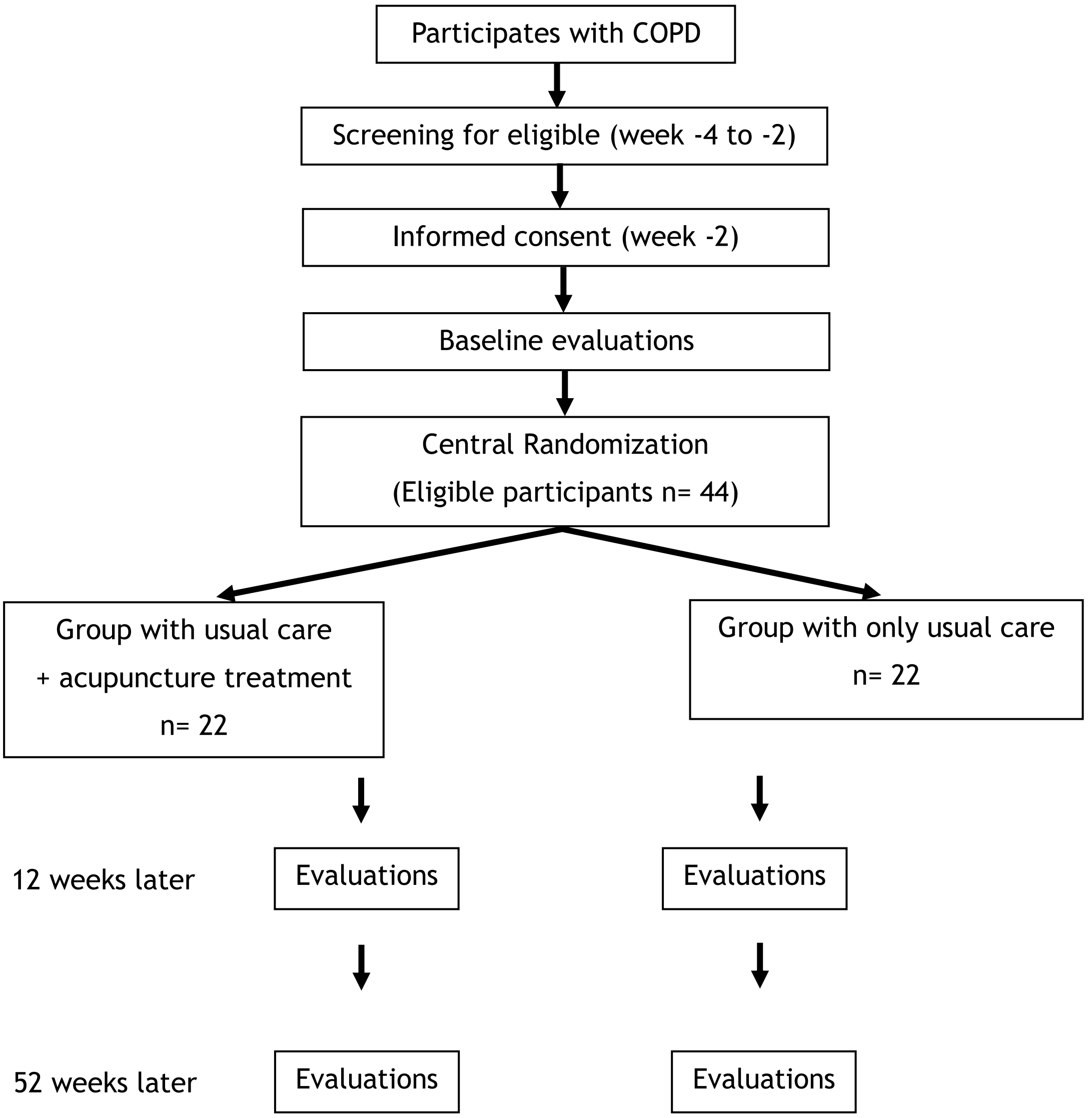

Data collection and Analysis

Material A-2 Study design schedule

Period	Baseline				Treatment		
Week	-4	-2	-1	0	1	12	52
Eligibility screening	⇒	⇒					
Informed consent		✓					
Randomization				✓			
Usual care					⇒	⇒	⇒
Acupuncture					⇒	⇒	⇒
Evaluations							
SGRQ			✓			✓	✓
6MWT			✓			✓	✓
Nutritional measures			✓			✓	✓
MMRC			✓			✓	✓
CAT			✓			✓	✓
BODE index			✓			✓	✓
Thorax excursion			✓			✓	✓
Precision lung function test			✓			✓	✓
IOS			✓			✓	✓
Respiratory muscle strength			✓			✓	✓
Inflammatory biomarkers			✓			✓	✓
Expectations for acupuncture			✓				
COPD dairy					✓	✓	✓

SGRQ : St. George's Respiratory Questionnaire, 6MWT: 6-minute walk test, MMRC: Modified Medical Research Council dyspnea scale, CAT: COPD Assessment Test, IOS: Impulse

Oscillation System, COPD: Chronic Obstructive Pulmonary Disease.

Material B GOLD Classification

Stage	Characteristics
0 :	Normal Spirometry, Chronic Symptoms (cough, sputum production)
I :	FEV1/FVC<70%, FEV1≧80%Predicted, With or without chronic symptoms
II :	FEV1/FVC<70%, 50%≦FEV1＜80%Predicted, With or without chronic symptoms
III :	FEV1/FVC<70%, 30%≦FEV1＜50%Predicted, With or without chronic symptoms
IV :	FEV1/FVC<70%, FEV1＜30%Predicted, or FEV1＜50%Predicted plus, chronic respiratory failure or right heart failure complication

FEV1: Forced expiratory volume in one second, FVC: Forced vital capacity, %FEV1: Percent predicted forced expiratory volume in one second.

Material C-1 Method of Acupuncture

Method of acupuncture treatment (acupuncture group)

The acupuncture points used in this clinical trial are selected from those reported effective for obstructive ventilator defects (COPD, bronchial asthma).

- **Lung Meridian**

1: Zhongfu (LU 1), 1 cun below from inside coracoid.
2: Taiyuan (LU 9), Palm wrist at radial artery.

- **Conception Vessel**

3: Guanyuan (CV 4), 3 cun below the navel.
4: Zhongwan (CV 12), 4 cun above the navel.

5: Shanzhong (CV 17), Front midline at height of 4th intercostal.

- **Large Intestine Meridian**

6: Futu (LI 18), 3 cun outside laryngeal prominence (in stemocleidomastoid).

- **Stomach Meridian**

7: Zusanli (ST 36), Intersection of tibial tuberosity inferior border and head of fibula inferior border.

- **Kidney Meridian**

8: Taixi (KI 3), Center of medial malleolus and calcaneal tendon.

- **Bladder Meridian**

9: Tianzhu (BL 10), Posterior cervical at the height of the second cervical spine spinous process superior margin, concavity of the outside trapezius.

10: Feishu (BL 13), 1.5 cun outside between the third and the fourth thoracic vertebrae spinous processes.

11: Pishu (BL 20), 1.5 cun outside between the 11th and the 12th lumbar vertebrae spinous processes.

12: Shenshu (BL 23), 1.5 cun outside between the second and the third lumbar vertebrae spinous processes.

A position of acupuncture point is commonly expressed by cun. One cun is about 3cm. Acupuncture needles used are stainless steel Japanese disposable needles (made by Seirin Co., Ltd), 40mm in length and 0.14 to 0.25mm in thickness (Figure 2).
After inserting an acupuncture needle into each acupuncture point and the patient feels the deqi, the needle is rotated in a direction for 3 minutes to keep the deqi.
Total treatment time is about 60 minutes. This procedure is performed on all acupuncture points.

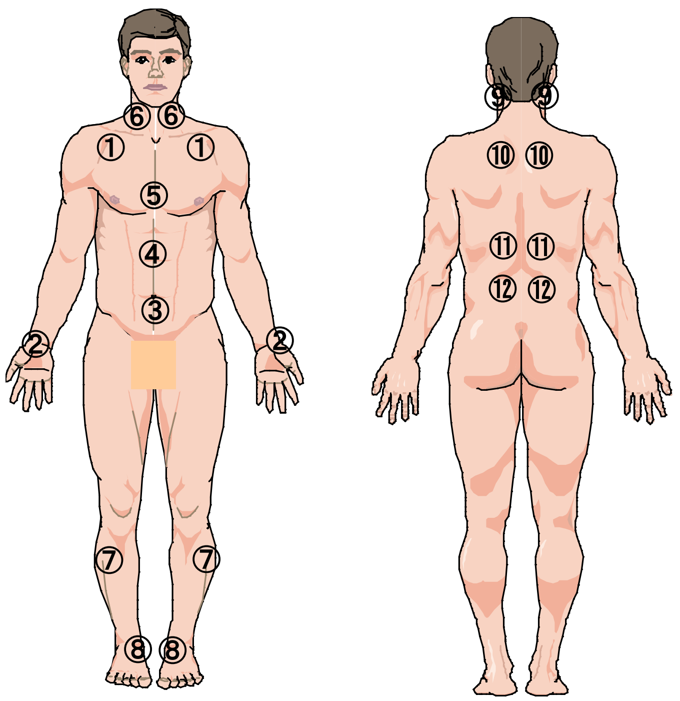

Figure 1: Acupuncture points used

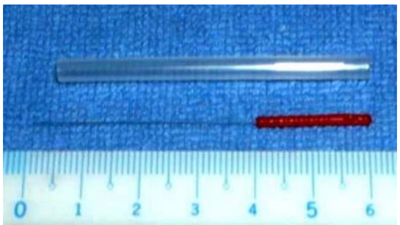

Figure 2-A: Acupuncture needle used
Material C-2 Method of Acupuncture (in table)

Acupoints	Location	Depth of insertion	How to insertion
Zhongfu (LU 1)	In the superior lateral part of the anterior thoracic wall, 1 cun below Yunmen(LU2), on the level with the 1st intercostal space, 6cun lateral to the anterior midline.	≦5mm	Insert vertically
Taiyuan (LU9)	At the radial end of the crease of the wrist, where the pulse of the radial artery is palpable.	10 to 20mm	Acupuncture needles are inserted horizontally into the skin from the periphery to the center.
Guanyuan (CV 4)	The lower abdomen on the anterior midline, 3 cun below the center of the umbilicus.	10 to 30mm	Insert vertically
Zhongwan (CV 12)	The upper abdomen on the anterior midline, 4 cun above the center of the umbilicus.	10 to 30mm	Insert vertically
Tanzhong (CV 17)	The chest on the anterior midline, level with the 4th intercostal space, at the midpoint of the line connecting both nipples.	10 to 20mm	Acupuncture needle is inserted at an angle of 30 degrees toward the sternum.
Futu (LI 18)	On the lateral side of the neck, beside laryngeal prominence, between the anterior and posterior borders of the sternocleidomastoid muscle.	10 to 20mm	Insert vertically
Zusanli (ST 36)	On the anterior lateral side of the leg, 3 cun below Dusi(ST35), one finger breadth from the anterior crest of the tibia.	10 to 30mm	Insert vertically
Taixi (KI 3)	On the medial side of the foot, posterior on the medial malleolus, in the depression between the tip of the medial malleolus and	10 to 20mm	Insert vertically

	Achilles tendon.		
Tianzhu (BL 10)	On the neck, in the depression of the lateral border of the trapezius muscle, 1.3 cun lateral to the midpoint of the posterior hairline.	10 to 30mm	Insert vertically
Feishu (BL 13)	On the back, below the spinous process of the 3rd thoracic vertebra, 1.5cun lateral to the posterior midline.	≦5 mm	Insert vertically
Pishu (BL 20)	In prone position, locate the point below the spinous process of the 11th thoracic vertebrae, about 1.5cun lateral to Jizhong.	5 to 10mm	Insert vertically
Shenshu (BL 23)	On the lower back, below the spinous process of the 2nd lumbar vertebra, 1.5 cun lateral to the posterior midline.	5 to 15mm	Insert vertically

Material D: Modified Borg Scale

0	Nothing at all
0.5	Very, Very slight
1	Very slight
2	Slight
3	
4	Somewhat severe
5	Severe
6	
7	Very severe
8	
9	
10	Very, very severe

Material E: Modified MRC Dyspnea Scale

Grade 0	There is shortness of breath only when exercising intensely.
Grade 1	There is shortness of breath when I walk on a flat road quickly or when I walk on a gentle uphill.
Grade 2	Because of shortness of breath, I walk on a flat road slower than a person

	of the same age or I sometimes stop due to shortness of breath while walking on a flat road at my own pace.
Grade 3	I stop due to shortness of breath when I walk for about 100 meters on a flat road.
Grade 4	I cannot get out of my house due to severe shortness of breath or there is shortness of breath when I change clothes.

Material F: Evaluation of Acute Aggravation

Major Item		
	(1)	Obvious worsening of dyspnea
	(2)	Obvious increase of sputum purulence
	(3)	Obvious increase of sputum quantity
Minor item		
	(1)	Upper respiratory tract infection lasting more than 5 days
	(2)	Fever without other obvious cause
	(3)	Increased wheezing
	(4)	Increased cough
	(5)	Respiratory rate or heart rate increased more than 20%

Material G: Organization Structure

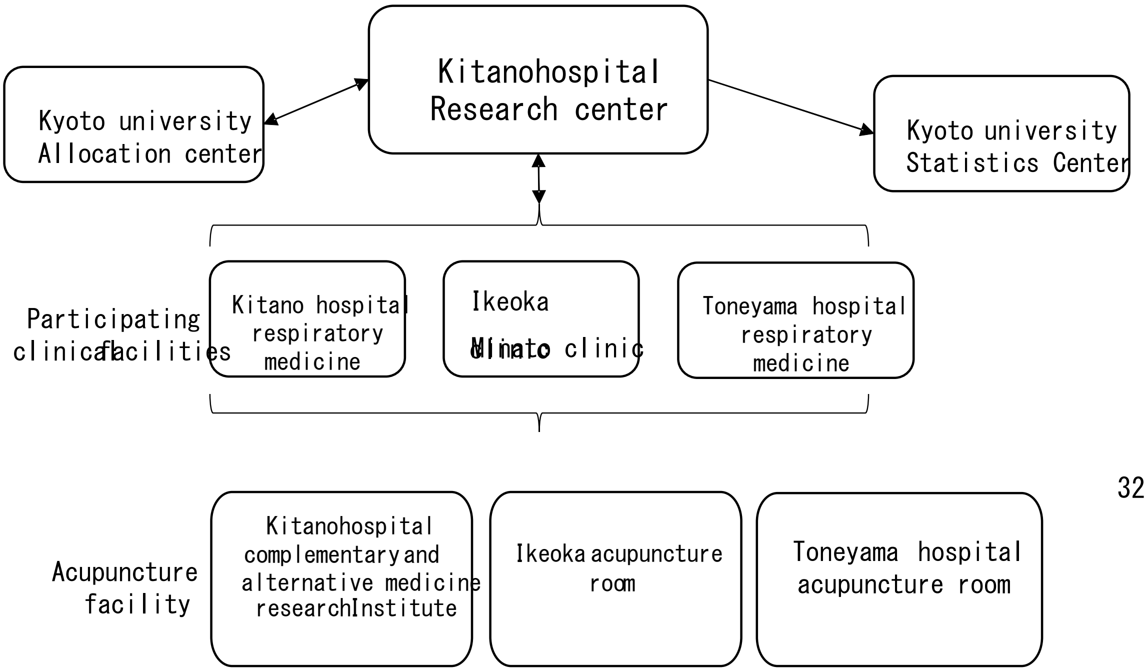

Supplement: Supplementary file 1 [file 00668-2024.SUPPLEMENT.pdf]
