# Supplementary material for: Effectiveness of a long-term acupuncture treatment in patients with COPD: a randomised controlled trial
Source: ERJ Open Res. 2025 May 19;11(3):00668-2024. doi: 10.1183/23120541.00668-2024 (PMC12086826; doi:10.1183/23120541.00668-2024)
Supplement: Supplementary file 2 [file 00668-2024.SUPPLEMENT2.pdf]

Effect of long term intervention with acupuncture on COPD: A randomized controlled trial.　Long term COPD Acupuncture Trial (LCAT)

Statistical analysis plan

Version 1.0

Written by Yoshimitsu T on July 17th, 2012(First version 1.0)

1. Preface

This is a statistical analysis plan for the Long term COPD Acupuncture Trial (LCAT) (hereinafter referred to as this study), which examines the long-term effects of acupuncture on Chronic Obstructive Pulmonary Disease (COPD).

The purpose and research plan of LCAT are described in the LCAT protocol.

The protocol stipulates that the main analysis of this study should be performed blindly, and the results performed blindly will report the results of groups A and B. This plan describes the analysis performed blindly.

The statistical analyst shall immediately perform the data analysis according to the contents described in this plan after the study is completed and the data sheets of all patients are received.

The statistician shall prepare an analysis report of the analysis carried out in accordance with this plan and submit it to the clinical researcher. Until this report is submitted, the statistician must not know which treatment was assigned to which group.

There may be a request for new data analysis after the statistician knows the allocation result. In such cases, the statistician shall carry out data analysis other than those described in this plan.

2. Outcome measures and observation items

Primary outcome measure, secondary outcome measures, other outcome measures, and observation items in this study are as shown below.

[Primary outcome measure]

- QOL Questionnaire: SGRQ (St. George 's Respiratory Questionnaire) Total domain value in Japanese version (sgrq_total) 【unit】

Compare the difference between the values one year later and at baseline.

[Secondary outcome measures]

- QOL Questionnaire: SGRQ (St. George 's Respiratory Questionnaire) Total domain value in Japanese version (sgrq_total) 【unit】

Compare the difference between the values 3 months later and at baseline.

- QOL questionnaire: Items excluding SGRQ Total domain (Symptom, Activity, Impact) (sgrq_symptom, sgrq_activity, sgrq_impact) 【unit】
- Dyspnea at the end of the 6-minute walking test Modified Borg Scale value (mbs_walk) 【unit】
- Lower limb fatigue at the end of the 6-minute walking test Modified Borg Scale value (mbs_fatigue) 【unit】

- Arterial oxygen saturation (SpO_2) (lowest value during 6-minute walking test) (lowest spo2) 【%】
- Highest pulse during walking (highest pulse during 6-minute walking test) 【bpm】
- Walking distance in 6-minute walking test (6mwd) 【m】

For all of the above, compare the difference between the values 3 months later and at baseline and between the values 1 year later and baseline.

[Other outcome measures]

- BMI（Body Mass Index）【kg/m^2】
- MEP (maximum expiratory pressure) mep【cmH_2O】
- MET (maximum expiratory time) met【sec】
- MIP (maximum inspiratory pressure) mip【cmH_2O】
- MIT (maximum inspiratory time) mit【sec】
- VC (vital capacity) vc【L】
- %vital capacity %vc【%】
- ERV (expiratory reserve volume) erv【L】
- IRV (inspiratory reserve volume) irv【L】
- IC (inspiratory capacity) ic【L】
- FVC (forced vital capacity) fvc【L】
- FEV (forced expiratory volume) in one second fev_1【L】
- %predicted forced expiratory volume in one second %fev_1【%】
- %predicted forced expiratory volume in one second -Gaensler %fev_1-G【%】
- PEF (peak expiratory flow) pef【L/sec】
- FRC (functional residual capacity) frc【L】
- RV (residual volume) rv【L】
- TLC (total lung capacity) tlc【L】
- Rv/tlc【%】
- DLCO (diffusing capacity of the lung for carbon monoxide) dlco【ml/min/mmHg】
- %diffusing capacity of the lung for carbon monoxide %dlco【%】
- adjusted diffusing capacity of the lung ‘dlco【ml/min/mmHg】
- adjusted %diffusing capacity of the lung ‘%dlco【%】
- DLCO/VA (diffusing capacity of the lung/alveolar volume) dlco/va【ml/min/mmHg/L】
- Z5 (Z at 5Hz) z5【kPa/(L/s)】

- R5 (R at 5Hz)　r5【kPa/(L/s)】
- R20 (R at 20Hz) r20【kPa/(L/s)】
- Diff R5-R20　r5-r20【kPa/(L/s)】
- X5 (X at 5Hz)　x5【kPa/(L/s)】
- AX　ax【kPa/L】
- PREALB (serum prealbumin) prealb【g/dl】
- ALB (serum albumin) alb【g/dl】
- HGB (hemoglobin) hgb【g/dl】
- IL6 (Interleukin-6)　il6【pg/mL】
- TNF (Tumor Necrosis Factor-α) tnfα【pg/mL】
- HSCRP (high sensitive C-reactive protein) hscrp【ng/mL】
- MMRC (modified MRC dyspnea scale) mmrc【unit】
- CAT (COPD Assessment Test) cat
- grasping power right grip_rt【kg】
- grasping power left grip_lt【kg】
- thorax excursion ribcage【cm】
- TSF (triceps skinfold thickness) tsf【mm】
- AC (arm circumference) ac【cm】
- AMC (arm muscle circumference) amc【cm】
- AMA (arm muscle area) ama【cm^2】
- BODE index　bode index【unit】
- acute aggravation exa_time【times】
- mild aggravation mild【times】
- moderate aggravation moderate【times】
- severe aggravation sever【times】
- unscheduled outpatient visits unscheduled【times】
- hospitalization admi_time　【times】
- 6MWTSPO2 (SpO_2 value per minute during 6-minute walking test) (at baseline) bl1 6mwt sop2【%】
- 6MWTSPO2 (SpO_2 value per minute during 6-minute walking test) (3 months later) 3m 6mwt sop2【%】
- 6MWTSPO2 (SpO_2 value per minute during 6-minute walking test) (1 year later) 1y 6mwt sop2【%】
- 6MWTPULS (pulse per minute during 6-minute walking test) (at baseline) bl1 6mwt pulse【bpm】
- 6MWTPULS (pulse per minute during 6-minute walking test) (3 months later)

3m 6mwt pulse【bpm】

・ 6MWTPULS (pulse per minute during 6-minute walking test) (1 year later) 1y 6mwt pulse【bpm】
・ For all of the above, compare the difference between the values 3 months later and at baseline and between the values 1 year later and baseline.

Observation items

・ smoking history
・ average number of cigarettes × years of smoking (bi)
・ age
・ height ht【cm】
・ weight wt【kg】
・ gender (1=male, 2=female)
・ GOLD criteria （gold）
・ use of home oxygen therapy （hot）（1=n, 2=yes）
・ Expectations for acupuncture on COPD: a 6-step Likert scale (Expecting highly, Expecting so much, Expecting a little, Indifferent, Not expecting much, Not expecting at all)

Database

item	variable name	property	note
patient ID	no	number	
group	Group	text	A：group A,<br>B：group B
drop out	Drop	number	1：no, 2：yes
age	age	number	
gender	gender	number	1：male,<br>2：female
smoking history	bi	number	
GOLD criteria at participation	gold	number	
use of home oxygen therapy	hot	number	1 : no, 2 : yes
Expectations for acupuncture	Expct	number	
height at BL	ht bs1	number	
height 3 months (3M) later	ht 3m	number	
height 1 year (1Y) later	ht 1y	number	
difference of height between Bl and 3M	ht A	number	

difference of height between Bl and 1Y	ht B	number	
weight at BL	wt bs1	number	
weight 3M later	wt 3m	number	
weight 1Y later	wt 1y	number	
difference of weight between Bl and 3M	wt A	number	
difference of weight between Bl and 1Y	wt B	number	
BMI at BL	bmi bs1	number	
BMI 3M later	bmi 3m	number	
BMI 1Y later	bmi 1y	number	
difference of BMI between Bl and 3M	bmi A	number	
difference of BMI between Bl and 1Y	bmi B	number	
MEP at BL	mep bs1	number	
MEP 3M later	mep 3m	number	
MEP 1Y later	mep 1y	number	
difference of MEP between Bl and 3M	mep A	number	
difference of MEP between Bl and 1Y	mep B	number	
MET at BL	met bs1	number	
MET 3M later	met 3m	number	
MET 1Y later	met 1y	number	
difference of MET between Bl and 3M	met A	number	
difference of MET between Bl and 1Y	met B	number	
MIP at BL	mip bs1	number	
MIP 3M later	mip 3m	number	
MIP 1Y later	mip 1y	number	
difference of MIP between Bl and 3M	mip A	number	
difference of MIP between Bl and 1Y	mip B	number	
MIT at BL	mit bs1	number	
MIT 3M later	mit 3m	number	
MIT 1Y later	mit 1y	number	
difference of MIT between Bl and 3M	mit A	number	
difference of MIT between Bl and 1Y	mit B	number	
VC at BL	vc bs1	number	
VC 3M later	vc 3m	number	
VC 1Y later	vc 1y	number	
difference of VC between Bl and 3M	vc A	number	
difference of VC between Bl and 1Y	vc B	number	

%VC at BL	%vc bs1	number	
%VC 3M later	%vc 3m	number	
%VC 1Y later	%vc 1y	number	
difference of %VC between Bl and 3M	%vc A	number	
difference of %VC between Bl and 1Y	%vc B	number	
ERV at BL	erv bs1	number	
ERV 3M later	erv 3m	number	
ERV 1Y later	erv 1y	number	
difference of ERV between Bl and 3M	erv A	number	
difference of ERV between Bl and 1Y	erv B	number	
IRV at BL	irv bs1	number	
IRV 3M later	irv_3m	number	
IRV 1Y later	irv 1y	number	
difference of IRV between Bl and 3M	irv A	number	
difference of IRV between Bl and 1Y	irv B	number	
IC at BL	ic bs1	number	
IC 3M later	ic 3m	number	
IC 1Y later	ic 1y	number	
difference of IC between Bl and 3M	ic A	number	
difference of IC between Bl and 1Y	ic B	number	
FVC at BL	fvc bs1	number	
FVC 3M later	fvc 3m	number	
FVC 1Y later	fvc 1y	number	
difference of FVC between Bl and 3M	fvc A	number	
difference of FVC between Bl and 1Y	fvc A	number	
FEV1 at BL	fev1 bs1	number	
FEV1 3M later	fev1 3m	number	
FEV1 1Y later	fev1 1y	number	
difference of FEV1 between Bl and 3M	fev1 A	number	
difference of FEV1 between Bl and 1Y	fev1 B	number	
%FEV1 at BL	%fev1 bs1	number	
%FEV1 3M later	%fev1 3m	number	
%FEV1 1Y later	%fev1 1y	number	
difference of %FEV1 between Bl and 3M	%fev1 A	number	
difference of %FEV1 between Bl and 1Y	%fev1 B	number	
FEV1% at BL	fev1% bs1	number	

FEV1% 3M later	fev1% 3m	number	
FEV1% 1Y later	fev1% 1y	number	
difference of FEV1% between Bl and 3M	fev1% A	number	
difference of FEV1% between Bl and 1Y	fev1% B	number	
PEF at BL	pef bs1	number	
PEF 3M later	pef 3m	number	
PEF 1Y later	pef 1y	number	
difference of PEF between Bl and 3M	pef A	number	
difference of PEF between Bl and 1Y	pef B	number	
FRC at BL	frc bs1	number	
FRC 3M later	frc 3m	number	
FRC 1Y later	frc 1y	number	
difference of FRC between Bl and 3M	frc A	number	
difference of FRC between Bl and 1Y	frc B	number	
RV at BL	rv bs1	number	
RV 3M later	rv 3m	number	
RV 1Y later	rv 1y	number	
difference of RV between Bl and 3M	rv A	number	
difference of RV between Bl and 1Y	rv B	number	
TLC at BL	tlc bs1	number	
TLC 3M later	tlc 3m	number	
TLC 1Y later	tlc 1y	number	
difference of TLC between Bl and 3M	tlc A	number	
difference of TLC between Bl and 1Y	tlc B	number	
RV/TLC at BL	rv/tlc bs1	number	
RV/TLC 3M later	rv/tlc 3m	number	
RV/TLC 1Y later	rv/tlc 1y	number	
difference of RV/TLC between Bl and 3M	rv/tlc A	number	
difference of RV/TLC between Bl and 1Y	rv/tlc B	number	
DLCO at BL	dlco bs1	number	
DLCO 3M later	dlco 3m	number	
DLCO 1Y later	dlco 1y	number	
difference of DLCO between Bl and 3M	dlco A	number	
difference of DLCO between Bl and 1Y	dlco B	number	
DLCO% at BL	%dlco bs1	number	
DLCO% 3M later	%dlco 3m	number	

DLCO% 1Y later	%dlco 1y	number	
difference of DLCO% between Bl and 3M	%dlco A	number	
difference of DLCO% between Bl and 1Y	%dlco B	number	
DLCO' at BL	dlco' bs1	number	
DLCO' 3M later	dlco' 3m	number	
DLCO' 1Y later	dlco' 1y	number	
difference of DLCO' between Bl and 3M	dlco' A	number	
difference of DLCO' between Bl and 1Y	dlco' B	number	
%DLCO' at BL	%dlco' bs1	number	
%DLCO' 3M later	%dlco' 3m	number	
%DLCO' 1Y later	%dlco' 1y	number	
difference of %DLCO' between Bl and 3M	%dlco' A	number	
difference of %DLCO' between Bl and 1Y	%dlco' B	number	
DLCO/VA at BL	dlco/va bs1	number	
DLCO/VA 3M later	dlco/va 3m	number	
DLCO/VA 1Y later	dlco/va 1y	number	
difference of DLCO/VA between Bl and 3M	dlco/va A	number	
difference of DLCO/VA between Bl and 1Y	dlco/va B	number	
Z5 at BL	z5 bs1	number	
Z5 3M later	z5 3m	number	
Z5 1Y later	z5 1y	number	
difference of Z5 between Bl and 3M	z5 A	number	
difference of Z5 between Bl and 1Y	z5 B	number	
R5 at BL	r5 bs1	number	
R5 3M later	r5 3m	number	
R5 1Y later	r5 1y	number	
difference of R5 between Bl and 3M	r5 A	number	
difference of R5 between Bl and 1Y	r5 B	number	
R20 at BL	r20 bs1	number	
R20 3M later	r20 3m	number	
R20 1Y later	r20 1y	number	
difference of R20 between Bl and 3M	r20 A	number	
difference of R20 between Bl and 1Y	r20 B	number	
R5-R20 at BL	r5-r20 bs1	number	
R5-R20 3M later	r5-r20 3m	number	

R5-R20 1Y later	r5-r20 1y	number	
difference of R5-R20 between Bl and 3M	r5-r20 A	number	
difference of R5-R20 between Bl and 1Y	r5-r20 B	number	
X5 at BL	x5 bs1	number	
X5 3M later	x5 3m	number	
X5 1Y later	x5 1y	number	
difference of X5 between Bl and 3M	x5 A	number	
difference of X5 between Bl and 1Y	x5 B	number	
AX5 at BL	ax5 bs1	number	
AX5 3M later	ax5 3m	number	
AX5 1Y later	ax5 1y	number	
difference of AX5 between Bl and 3M	ax5 A	number	
difference of AX5 between Bl and 1Y	ax5 B	number	
PREALB at BL	prelab bs1	number	
PREALB 3M later	prealb 3m	number	
PREALB 1Y later	prealb 1y	number	
difference of PREALB between Bl and 3M	prealb A	number	
difference of PREALB between Bl and 1Y	prealb B	number	
ALB at BL	alb bs1	number	
ALB 3M later	alb 3m	number	
ALB 1Y later	alb 1y	number	
difference of ALB between Bl and 3M	alb A	number	
difference of ALB between Bl and 1Y	alb B	number	
HGB at BL	hgb bs1	number	
HGB 3M later	hgb 3m	number	
HGB 1Y later	hgb 1y	number	
difference of HGB between Bl and 3M	hgb A	number	
difference of HGB between Bl and 1Y	hgb B	number	
IL6 at BL	il6 bs1	number	
IL6 3M later	il6 3m	number	
IL6 1Y later	il6 1y	number	
difference of IL6 between Bl and 3M	il6 A	number	
difference of IL6 between Bl and 1Y	il6 B	number	
TNF at BL	tnf bs1	number	
TNF 3M later	tnf 3m	number	
TNF 1Y later	tnf 1y	number	

difference of TNF between Bl and 3M	tnf A	number	
difference of TNF between Bl and 1Y	tnf B	number	
HSCRP at BL	hscrp bs1	number	
HSCRP 3M later	hscrp 3m	number	
HSCRP 1Y later	hscrp 1y	number	
difference of HSCRP between Bl and 3M	hscrp A	number	
difference of HSCRP between Bl and 1Y	hscrp B	number	
SGRQSymp at BL	sgrq_symptom b1	number	
SGRQSymp 3M later	sgrq_symptom 3m	number	
SGRQSymp 1Y later	sgrq_symptom 1y	number	
difference of SGRQSymp between Bl and 3M	sgrq_symptom A	number	
difference of SGRQSymp between Bl and 1Y	sgrq_symptom B	number	Secondary outcome
SGRQACT at BL	sgrq_activity bs1	number	
SGRQACT 3M later	sgrq_activity 3m	number	
SGRQACT 1Y later	sgrq_activity 1y	number	
difference of SGRQACT between Bl and 3M	sgrq_activity A	number	
difference of SGRQACT between Bl and 1Y	sgrq_activity B	number	Secondary outcome
SGRQIMP at BL	sgrq_impact bs1	number	
SGRQIMP 3M later	sgrq_impact 3m	number	
SGRQIMP 1Y later	sgrq_impact 1y	number	
difference of SGRQIMP between Bl and 3M	sgrq_impact A	number	
difference of SGRQIMP between Bl and 1Y	sgrq_impact B	number	Secondary outcome
SGRQTOTA at BL	sgrq_total bs1	number	
SGRQTOTA 3M later	sgrq_total 3m	number	
SGRQTOTA 1Y later	sgrq_total 1y	number	
difference of SGRQTOTA between Bl and 3M	sgrq_total A	number	
difference of SGRQTOTA between Bl and 1Y	sgrq_total B	number	Main outcome
CAT at BL	cat bs1	number	

CAT 3M later	cat 3m	number	
CAT 1Y later	cat 1y	number	
difference of CAT between Bl and 3M	cat A	number	
difference of CAT between Bl and 1Y	cat B	number	
MBSW at BL	mbs_walk bs1	number	
MBSW 3M later	mbs_walk 3m	number	
MBSW 1Y later	mbs_walk 1y	number	
difference of MBSW between Bl and 3M	mbs_walk A	number	
difference of MBSW between Bl and 1Y	mbs_walk B	number	Secondary outcome
MBSF at BL	mbs_fatigue bs1	number	
MBSF 3M later	mbs_fatigue 3m	number	
MBSF 1Y later	mbs_fatigue 1y	number	
difference of MBSF between Bl and 3M	mbs_fatigue A	number	
difference of MBSF between Bl and 1Y	mbs_fatigue B	number	Secondary outcome
6MWD at BL	6mwd bs1	number	
6MWD 3M later	6mwd 3m	number	
6MWD 1Y later	6mwd 1y	number	
difference of 6MWD between Bl and 3M	6mwd A	number	
difference of 6MWD between Bl and 1Y	6mwd B	number	Secondary outcome
LOWSPO2 at BL	lowest spo2 bs1	number	
LOWSPO2 3M later	lowest spo2 3m	number	
LOWSPO2 1Y later	lowest spo2 1y	number	
difference of LOWSPO2 between Bl and 3M	lowest spo2 A	number	
difference of LOWSPO2 between Bl and 1Y	lowest spo2 B	number	Secondary outcome
HIGHPUL at BL	hightest pulse bs1	number	
HIGHPUL 3M later	hightest pulse 3m	number	
HIGHPUL 1Y later	hightest pulse 1y	number	
difference of HIGHPUL between Bl and 3M	hightest pulse A	number	
difference of HIGHPUL between Bl and 1Y	hightest pulse B	number	Secondary outcome

MMRC at BL	mmrc bs1	number	
MMRC 3M later	mmrc 3m	number	
MMRC 1Y later	mmrc 1y	number	
difference of MMRC between Bl and 3M	mmrc A	number	
difference of MMRC between Bl and 1Y	mmrc B	number	
RTGRIP at BL	grip_rt bs1	number	
RTGRIP 3M later	grip_rt 3m	number	
RTGRIP 1Y later	grip_rt 1y	number	
difference of RTGRIP between Bl and 3M	grip_rt A	number	
difference of RTGRIP between Bl and 1Y	grip_rt B	number	
LTGRIP at BL	grip_lt bs1	number	
LTGRIP 3M later	grip_lt 3m	number	
LTGRIP 1Y later	grip_lt 1y	number	
difference of LTGRIP between Bl and 3M	grip_lt A	number	
difference of LTGRIP between Bl and 1Y	grip_lt B	number	
RIB at BL	ribcage bs1	number	
RIB 3M later	ribcage 3m	number	
RIB 1Y later	ribcage 1y	number	
difference of RIB between Bl and 3M	ribcage A	number	
difference of RIB between Bl and 1Y	ribcage B	number	
TSF at BL	tsf bs1	number	
TSF 3M later	tsf 3m	number	
TSF 1Y later	tsf 1y	number	
difference of TSF between Bl and 3M	tsf A	number	
difference of TSF between Bl and 1Y	tsf B	number	
AC at BL	ac bs1	number	
AC 3M later	ac 3m	number	
AC 1Y later	ac 1y	number	
difference of AC between Bl and 3M	ac A	number	
difference of AC between Bl and 1Y	ac B	number	
AMC at BL	amc bs1	number	
AMC 3M later	amc 3m	number	
AMC 1Y later	amc 1y	number	
difference of AMC between Bl and 3M	amc A	number	
difference of AMC between Bl and 1Y	amc B	number	
AMA at BL	ama bs1	number	

AMA 3M later	ama 3m	number	
AMA 1Y later	ama 1y	number	
difference of AMA between Bl and 3M	ama A	number	
difference of AMA between Bl and 1Y	ama B	number	
BODE at BL	bodeindex bs1	number	
BODE 3M later	bodeindex 3m	number	
BODE 1Y later	bodeindex 1y	number	
difference of BODE between Bl and 3M	bodeindex A	number	
difference of BODE between Bl and 1Y	bodeindex B	number	
acute aggravation	exa_time	number	Count data
mild aggravation	mild	number	Count data
moderate aggravation	moderate	number	Count data
severe aggravation	sever	number	Count data
unscheduled outpatient visits	unscheduled	number	Count data
hospitalization	admission admi_time	number	Count data
6MWTSPO2 (at baseline) at rest	bl1 6mwt spo2 rest	number	
6MWTSPO2 (at baseline) 1min	bl1 6mwt spo2 1min	number	
6MWTSPO2 (at baseline) 2min	bl1 6mwt spo2 2min	number	
6MWTSPO2 (at baseline) 3min	bl1 6mwt spo2 3min	number	
6MWTSPO2 (at baseline) 4min	bl1 6mwt spo2 4min	number	
6MWTSPO2 (at baseline) 5min	bl1 6mwt spo2 5min	number	
6MWTSPO2 (at baseline) 6min	bl1 6mwt spo2 6min	number	
6MWTSPO2 (3months later) at rest	3m 6mwt spo2 rest	number	
6MWTSPO2 (3months later) 1min	3m 6mwt spo2 1min	number	
6MWTSPO2 (3months later) 2min	3m 6mwt spo2 2min	number	
6MWTSPO2 (3months later) 3min	3m 6mwt spo2 3min	number	
6MWTSPO2 (3months later) 4min	3m 6mwt spo2 4min	number	
6MWTSPO2 (3months later) 5min	3m 6mwt spo2 5min	number	
6MWTSPO2 (3months later) 6min	3m 6mwt spo2 6min	number	
6MWTSPO2 (1year later) at rest	1y 6mwt spo2 rest	number	
6MWTSPO2 (1year later) 1min	1y 6mwt spo2 1min	number	
6MWTSPO2 (1year later) 2min	1y 6mwt spo2 2min	number	
6MWTSPO2 (1year later) 3min	1y 6mwt spo2 3min	number	
6MWTSPO2 (1year later) 4min	1y 6mwt spo2 4min	number	
6MWTSPO2 (1year later) 5min	1y 6mwt spo2 5min	number	
6MWTSPO2 (1year later) 6min	1y 6mwt spo2 6min	number	

6MWTPULS (at baseline) at rest	bl1 6mwt pulse rest	number	
6MWTPULS (at baseline) 1min	bl1 6mwt pulse 1min	number	
6MWTPULS (at baseline) 2min	bl1 6mwt pulse 2min	number	
6MWTPULS (at baseline) 3min	bl1 6mwt pulse 3min	number	
6MWTPULS (at baseline) 4min	bl1 6mwt pulse 4min	number	
6MWTPULS (at baseline) 5min	bl1 6mwt pulse 5min	number	
6MWTPULS (at baseline) 6min	bl1 6mwt pulse 6min	number	
6MWTPULS (3months later) at rest	3m 6mwt pulse rest	number	
6MWTPULS (3months later) 1min	3m 6mwt pulse 1min	number	
6MWTPULS (3months later) 2min	3m 6mwt pulse 2min	number	
6MWTPULS (3months later) 3min	3m 6mwt pulse 3min	number	
6MWTPULS (3months later) 4min	3m 6mwt pulse 4min	number	
6MWTPULS (3months later) 5min	3m 6mwt pulse 5min	number	
6MWTPULS (3months later) 6min	3m 6mwt pulse 6min	number	
6MWTPULS (1year later) at rest	1y 6mwt pulse rest	number	
6MWTPULS (1year later) 1min	1y 6mwt pulse 1min	number	
6MWTPULS (1year later) 2min	1y 6mwt pulse 2min	number	
6MWTPULS (1year later) 3min	1y 6mwt pulse 3min	number	
6MWTPULS (1year later) 4min	1y 6mwt pulse 4min	number	
6MWTPULS (1year later) 5min	1y 6mwt pulse 5min	number	
6MWTPULS (1year later) 6min	1y 6mwt pulse 6min	number	

3. Analysis target population

In this study, two analysis target populations, FAS (Full Analysis Set) and PPS (Per Protocol Set), and data analysis is performed for each analysis target population.

The main analysis target population in this study is FAS.

The definitions of FAS and PPS in this study are defined as follows.

FAS: Population of all assigned patients, except those who were not evaluated at the final stage due to the dropout specified in the protocol.

PPS: Patients who achieved 40 or more acupuncture treatments in one year, excluding dropout patients.

Also, since the data analysis in this study is performed blindly, the clinical researchers determine whether or not the factors meet the conditions considered at the time of planning, and statistician shall only be informed of whether or not the factors meet the conditions considered at the time of

planning.

4. Method of comparison between groups

For the primary and secondary outcomes, comparison between groups is performed. For comparison between groups, analysis of covariance (ANCOVA) is performed for the difference in before and after treatments, including the group, pretreatment value, and layer used for allocation (GOLD classification), to calculate the parameter of the group with 95% confidence interval.

The model of ANCOVA is as follows.

$$Y_{\mathrm{diff}} = \beta_0 + \beta_1 x_{\mathrm{G}} + \beta_2 x_{\mathrm{B}} + \beta_3 x_{\mathrm{L1}} + \beta_4 x_{\mathrm{L2}} + \mathrm{error}$$

where

Y_{diff} is the difference between the baseline measurement and the measurement after 1 year (or 3 months) of the primary or the secondary outcomes,

x_{G} is the variable indicating the group (A or B).

x_{B} is the baseline value of measurements.

x_{L1} and x_{L2} are the variables indicating the layer at the time of allocation (GOLD classification: 2, 3, 4) (reference is GOLD classification 2), and

β_0、β_1、β_2、β_3、β_4 are parameters.

The results of the comparison between groups are shown by the β_1 estimates obtained by applying the above model and their 95% confidence intervals.

Not including 0 in the 95% confidence interval is synonymous with being statistically significant at the 5% significance level.

For the change from baseline in other outcome measures, an independent sample t-test is use. Continuous variables and ordinal variables are shown as the means (standard deviation), and their significant differences are shown by 95% confidence intervals (CI) and p-values (significance level 0.05). Count variables are shown as the number of counts and percentages, and X2 test or Fisher's exact test results are shown with p-values (significance level 0.05). For arterial oxygen saturation and pulse changes over time in the 6-minute walk test, a generalized linear mixed-effects model is used to analyze the differences between baseline, 12-week, and 52-week changes. The model treats group, time, and group * time interactions as fixed effects, and individuals as random effects by maximum likelihood method.

In addition, as a post hoc sensitivity analysis, analysis of covariance will be performed

with multiple imputation for the primary and secondary outcome measures, including missing data for dropouts. For dropout cases, multiple imputation is performed assuming MAR (Missing At Random), and when doing so, variables that exhibit a correlation (>0.5) with the baseline values of each evaluation item and the missing variables are used as auxiliary variables. When imputing missing values by multiple imputation, 100 data sets are generated and performed.

5. Handling of missing values

If there is a missing value, the patient ID and the reason for the missing data will be recorded. Also, for drop out and death cases, data will not be supplemented and will be excluded from the first analysis. However, if missing data occur, a post-hoc analysis is performed to complete the data and carry out the analysis.

6. Variable transformation

Depending on the indicator, the distribution of the obtained data may not be appropriately summarized by mean and standard deviation. In such cases, we will report a summary of the values that have undergone variable transformation.

When variable transformation is performed, the transformation formula and the reason for it shall be specified.

7. Report format of the data analysis results

The report format is described below. The following reports will be prepared for FAS and PPS.

7.1 Numbers of patients registered, assigned, and evaluated

To show the number of patients, make a diagram like the one below and enter the numbers.

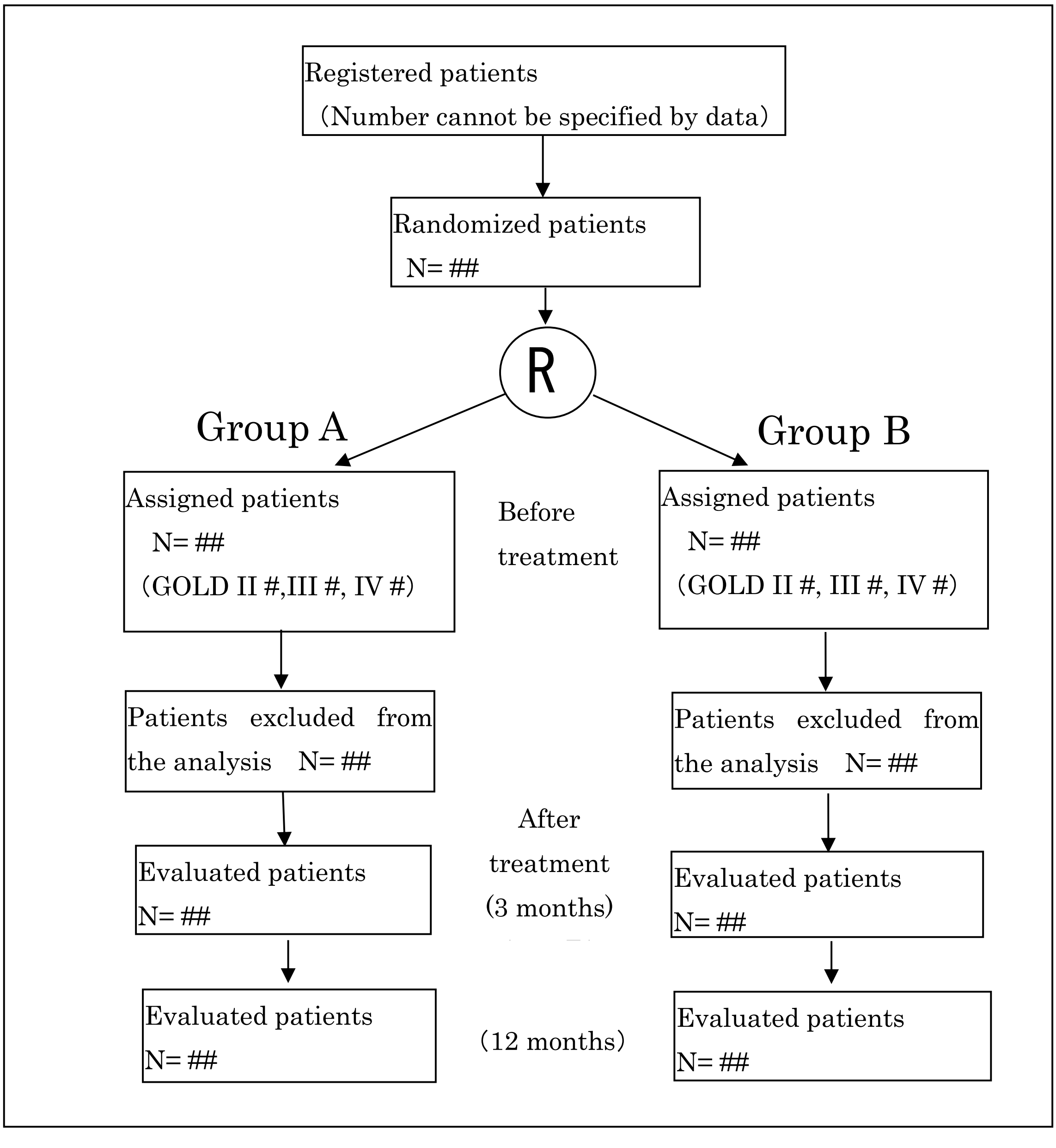

Registered patients
(Number cannot be specified by data)
Randomized patients
N= ##
R
Group A
Group B
Assigned patients
N= ##
(GOLD II #,III #, IV #)
Before
treatment
Assigned patients
N= ##
(GOLD II #, III #, IV #)
Patients excluded from the analysis N= ##
Patients excluded from the analysis N= ##
After
treatment
(3 months)
Evaluated patients
N= ##
Evaluated patients
N= ##
(12 months)
Evaluated patients
N= ##
Evaluated patients
N= ##

A table like the one below summarizes the patients with missing values.

Group A		Group B	
Patient ID（age）	reason	Patient ID（age）	reason
@	@	@	@
@	@	@	@
@	@	@	@
@	@	@	@
@	@	@	@

7.2 Distribution of patient background

Create a table like the one below to show the distribution of patient background.

Image of the distribution of patient background

		Group A N=@		Group B N=@	
age		XX.X	(SD XX.X)	XX.X	(SD XX.X)
sex	Male	@	(@%)	@	(@%)
	Female	@	(@%)	@	(@%)
GOLD	Stage II	@	(@%)	@	(@%)
	Stage III	@	(@%)	@	(@%)
	Stage IV	@	(@%)	@	(@%)
Smoking history		XX.X	(SD XX.X)	XX.X	(SD XX.X)
MMRC grade at entry		XX.X	(SD XX.X, range X-X)	XX.X	(SD XX.X, range X-X)

7.3 Presentation of the results of the primary and secondary outcomes

For the primary and secondary outcomes, create the following figures and tables.

- Scatter plot of measured values before treatment (at BL) and after treatment (1 year, 3 months).
- Table of basic statistics of pre-treatment, post-treatment, and differences.
- Comparison results (parameters and 95% confidence intervals).

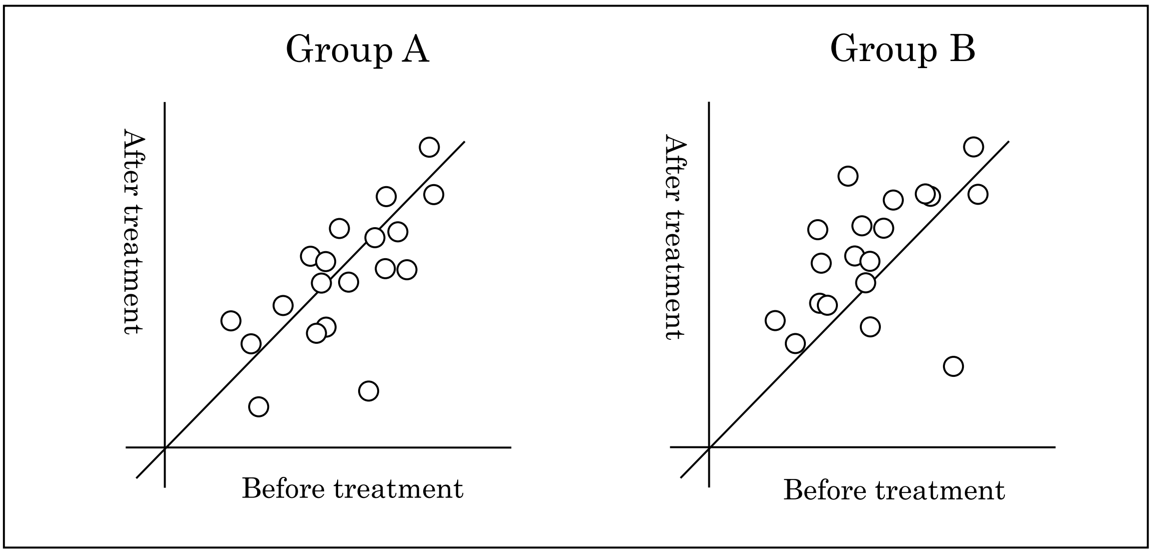

Image of scatter plot of measured values before and after treatment (color-coded by GOLD classification)

Mean and standard deviation of each group

GOLD	Group	at BL mean±SD（n）	3 months later mean±SD（n）	1 year later mean±SD（n）	3 mos－BL mean±SD（n）	1year－BL mean±SD（n）
Stage II	Group A	XX.X±XX.X（@）	XX.X±XX.X（@）	XX.X±XX.X（@）	XX.X±XX.X（@）	XX.X±XX.X（@）
	Group B	XX.X±XX.X（@）	XX.X±XX.X（@）	XX.X±XX.X（@）	XX.X±XX.X（@）	XX.X±XX.X（@）
Stage III	Group A	XX.X±XX.X（@）	XX.X±XX.X（@）	XX.X±XX.X（@）	XX.X±XX.X（@）	XX.X±XX.X（@）
	Group B	XX.X±XX.X（@）	XX.X±XX.X（@）	XX.X±XX.X（@）	XX.X±XX.X（@）	XX.X±XX.X（@）
Stage IV	Group A	XX.X±XX.X（@）	XX.X±XX.X（@）	XX.X±XX.X（@）	XX.X±XX.X（@）	XX.X±XX.X（@）
	Group B	XX.X±XX.X（@）	XX.X±XX.X（@）	XX.X±XX.X（@）	XX.X±XX.X（@）	XX.X±XX.X（@）

Comparison between groups

Number of cases evaluated	Therapeutic effect of Group B on Group A	P-value	95% confidence interval
@	XX.X	XX.X	[XX.X, XX.X]

7.4 Presentation of the results of other evaluation items

For other evaluation items, create following tables. When variable transformation is performed, it will be noted. In the FAS analysis, the missing data is calculated without data complementation.

Continuous variable

	Group	at BL mean±SD (n)	3 months later mean±SD (n)	1 year later mean±SD (n)	3 mos－1year mean±SD (n)	1 year－BL 95%CI
BMI	A	XX.X±XX.X (@)	XX.X±XX.X (@)	XX.X±XX.X (@)	XX.X±XX.X (@)	[XX.X, XX.X]
	B	XX.X±XX.X (@)	XX.X±XX.X (@)	XX.X±XX.X (@)	XX.X±XX.X (@)	
MMRC	A	XX.X±XX.X (@)	XX.X±XX.X (@)	XX.X±XX.X (@)	XX.X±XX.X (@)	
	B	XX.X±XX.X (@)	XX.X±XX.X (@)	XX.X±XX.X (@)	XX.X±XX.X (@)	
$PaCO_2$	A	XX.X±XX.X (@)	XX.X±XX.X (@)	XX.X±XX.X (@)	XX.X±XX.X (@)	
	B	XX.X±XX.X (@)	XX.X±XX.X (@)	XX.X±XX.X (@)	XX.X±XX.X (@)	
VC	A	XX.X±XX.X (@)	XX.X±XX.X (@)	XX.X±XX.X (@)	XX.X±XX.X (@)	
	B	XX.X±XX.X (@)	XX.X±XX.X (@)	XX.X±XX.X (@)	XX.X±XX.X (@)	
%VC	A	XX.X±XX.X (@)	XX.X±XX.X (@)	XX.X±XX.X (@)	XX.X±XX.X (@)	
	B	XX.X±XX.X (@)	XX.X±XX.X (@)	XX.X±XX.X (@)	XX.X±XX.X (@)	
TV	A	XX.X±XX.X (@)	XX.X±XX.X (@)	XX.X±XX.X (@)	XX.X±XX.X (@)	
	B	XX.X±XX.X (@)	XX.X±XX.X (@)	XX.X±XX.X (@)	XX.X±XX.X (@)	
ERV	A	XX.X±XX.X (@)	XX.X±XX.X (@)	XX.X±XX.X (@)	XX.X±XX.X (@)	

	B	XX.X±XX.X (@)	XX.X±XX.X (@)	XX.X±XX.X (@)	XX.X±XX.X (@)	
IC	A	XX.X±XX.X (@)	XX.X±XX.X (@)	XX.X±XX.X (@)	XX.X±XX.X (@)	
	B	XX.X±XX.X (@)	XX.X±XX.X (@)	XX.X±XX.X (@)	XX.X±XX.X (@)	
FRC	A	XX.X±XX.X (@)	XX.X±XX.X (@)	XX.X±XX.X (@)	XX.X±XX.X (@)	
	B	XX.X±XX.X (@)	XX.X±XX.X (@)	XX.X±XX.X (@)	XX.X±XX.X (@)	
···	A					
	B					

Count data

		3 months later			1 year later		
		Group A n(%)	Group B n(%)	P-value	Group A n(%)	Group B n(%)	P-value
Aggravation (mild-)	0	@(X.X%)	@(X.X%)	0.XX	@(X.X%)	@(X.X%)	0.XX
	1	@(X.X%)	@(X.X%)		@(X.X%)	@(X.X%)	
	2	@(X.X%)	@(X.X%)		@(X.X%)	@(X.X%)	
	3	@(X.X%)	@(X.X%)		@(X.X%)	@(X.X%)	
	···						
Aggravation (moderate-)							
Aggravation (severe-)							
Unscheduled outpatient visits							
Hospitalization							

End of the document.
