# Supplementary material for: Effectiveness of a long-term acupuncture treatment in patients with COPD: a randomised controlled trial
Source: ERJ Open Res. 2025 May 19;11(3):00668-2024. doi: 10.1183/23120541.00668-2024 (PMC12086826; doi:10.1183/23120541.00668-2024)
Supplement: Supplementary file 3 [file 00668-2024.SUPPLEMENT3.pdf]

Supplementary Material

Table of Contents

Table 1 Allocation results for each institution.. 2
Table 2 Results for training in acupuncture techniques. 3
Table 3 A generalized linear mixed effects model 4
Table 4 Changes in the muscle strength 5
Table 5 Thorax mobility 6
Table 6 Exacerbation 7
Table 7 mMRC. 8
Table 8 CAT 9
Table 9 BODE index 10
Table 10 Impulse Oscillation System 11

Table 1 Allocation results for each institution.

Institution	UG	AG
A General hospital	8	10
B General hospital	3	2
C Clinic	6	6
D Clinic	4	6

values indicate number of examples.
AG: Acupuncture group, UG: Usual care group.

Table 2 Results for training in acupuncture techniques.

Acupuncturists	after a week	after 2 weeks	after 3 weeks	after 4 weeks	after 5 weeks
A	23	24	24	25	25
B	24	25	24	25	25
C	22	24	24	25	25
D	23	24	24	25	25

・Acupuncturists participating in the study must meet the acceptance criteria within five weeks of receiving weekly evaluations from their instructors.
The evaluation consists of five items, with five criteria each:
Q1: Is the location of the acupuncture point, correct?
Q2: Is the order of needle insertion appropriate?
Q3: Is the needle insertion technique appropriate?
Q4: Is the needle rotation technique correct?
Q5: Is the deqi level appropriate?
・The evaluation criteria require a minimum total score of 20 points to pass.
The criteria are rated on a scale of 1 to 5, with the following meanings:
1: Not at all appropriate
2: Slightly appropriate
3: Fairly appropriate
4: Very appropriate
5: Extremely appropriate

Acupuncturists;
A: This acupuncturist had 17 years of continuous clinical experience.
B: This acupuncturist had 13 years of continuous clinical experience.
C: This acupuncturist had 12 years of continuous clinical experience.
D: This acupuncturist had 10 years of continuous clinical experience

Table 3 A generalized linear mixed effects model

	After 12 weeks			After 52 weeks		
	Parameter estimate	95% CI	*P* Value	Parameter estimate	95% CI	*P* Value
SpO_2						
time	-0.0	-0.2 to 0.2	0.96	-0.3	-0.6 to -0.0	0.022
group	1.8	0.6 to 3.1	0.01	2.3	1.3 to 3.3	<0.001
group x time	0.3	-0.1 to 0.6	0.10	0.6	0.2 to 1.0	0.001
Pulse						
time	0.9	-0.1 to 2.0	0.09	0.7	-0.5 to 1.8	0.253
group	-5.9	-11.6 to -0.1	0.05	-15.4	-22.5 to -8.3	<0.001
group x time	-0.8	-2.4 to 0.7	0.28	-0.8	-2.4 to 0.9	0.347

Regarding SpO_2, the difference between the two groups were 1.8% (95% CI 0.6 to 3.1) at rest before waking and 0.3% (-0.1 to 0.6) per minute at the week 12, and 2.3% (1.3 to 3.3) at rest and 0.6% (0.2 to 1.0) per minutes at week 52. Regarding pulse, the difference between the two groups were -5.9 bpm (95% CI -11.6 to -0.1) at rest and -0.8 bpm (-2.4 to 0.7) per minute at week 12, and -15.4 (-22.5 to -8.3) at rest and 0.8bpm (-2.4 to 0.9) per minutes at week 52.

Table 4 Changes in the muscle strength

Muscle Strength	Baseline	After 12 weeks	After 52 weeks	Change from baseline to after 12 weeks	Mean difference 95% CI	Change from baseline to after 52 weeks	Mean difference 95% CI
**Grasping power Right (kg)**							
UG	27.8 [6.0]	27.2 [6.4]	26.4 [6.1]	-0.7 [2.5]	2.6 [1.1 to 4.0]	-1.4 [2.2]	5.5 [3.9 to 7.1]
AG	27.5 [6.7]	29.4 [5.8]	31.6 [5.5]	1.9 [2.2]		4.1 [2.8]	
**Grasping power Left (kg)**							
UG	26.6 [6.5]	25.7 [6.1]	25.3 [6.0]	-0.9 [2.2]	2.3 [1.0 to 3.7]	-1.3 [2.3]	5.4 [3.6 to 7.2]
AG	26.5 [6.8]	28.0 [6.5]	30.6 [5.4]	1.5 [2.0]		4.1 [3.1]	
**Respiratory Muscle Strength**							
**MEP (cmH_2O)**							
UG	63.5 [28.6]	56.8 [22.3]	52.6 [21.0]	-6.7 [17.7]	39.9 [29.0 to 50.8]	-10.9 [24.1]	61.4 [45.6 to 77.1]
AG	61.1 [27.2]	94.3 [33.5]	111.6 [44.4]	33.2 [16.3]		50.5 [25.2]	
**MIP (cmH2O)**							
UG	66.5 [26.4]	62.3 [29.2]	57.5 [27.7]	-4.2 [10.0]	14.0 [7.4 to 20.7]	-9.0 [12.8]	21.6 [13.5 to 29.6]
AG	63.3 [25.0]	73.2 [26.9]	75.9 [26.4]	9.9 [10.8]		12.6 [12.4]	

UG, usual care group; AG, acupuncture group; MEP, maximum expiratory mouth pressure; MIP, maximum inspiratory mouth pressure; 95%CI, 95% confidence interval.

Table 5 Thorax mobility

Thorax mobility (cm)	Baseline	After 12 weeks	After 52 weeks	Change from baseline to after 12 weeks	Mean difference 95% CI	Change from baseline to after 52 weeks	Mean difference 95% CI
UG	2.8 [1.3]	2.9 [1.1]	2.6 [1.0]	0.1 [1.2]	2.6 [1.6 to 3.5]	-0.3 [1.1]	5.2 [3.8 to 6.5]
AG	3.4 [1.4]	6.1 [2.1]	8.4 [2.6]	2.7 [1.7]		4.9 [2.8]	

Values are mean [SD] unless otherwise stated.
The number of patients in each case was 20 in UG and 20 in AG.

Thorax mobility

The thorax mobility improved from 3.4 [1.4] at baseline to 6.1 [2.1] at week 12, to 8.4 [2.6] at week 52 in the AG. The difference in the thorax mobility in the AG (2.7 [1.7] at week 12, 4.9 [2.8] at week 52) were statistically significant by t-test compared with that in the UG (0.1 [1.2] at week 12, -0.3 [1.1] at week 52) (mean difference 2.6, 95% CI 1.6 to 3.5 at week 12, 5.2 [3.8 to 6.5] at week 52).

AG: acupuncture group, UG: Usual care group, 95%CI: 95% confidence interval.

Table 6 Exacerbation

**Exacerbation**	Number of exacerbations	UG n(%)	AG n(%)	*p* value
Mild	0	9(45)	8(40)	0.631
	1	8(40)	8(40)	
	2	2(10)	4(20)	
	3	1(5)	0(0)	
Moderate	0	7(35)	14(70)	0.009
	1	6(30)	6(30)	
	2	7(35)	0(0)	
Severe	0	13(65)	19(95)	0.018
	1	7(35)	1(5)	
Overall				0.052
Number of unscheduled outpatients	0	7(35)	15(75)	0.013
	1	13(65)	4(20)	
	2	0(0)	1(5)	
Number of hospitalizations	0	17(85)	20(100)	0.072
	1	3(15)	0(0)	

During the study period (52 weeks), the number of moderate or high exacerbations was significantly lower in the AG than in the UG, and the number of unscheduled hospital visits associated with exacerbation was also significantly lower. However, there was no significant difference in the number of hospitalizations associated with exacerbations (three patients in the UG and no patients in the AG).

Count variables are shown as the number of counts and percentages. Chi-square test or Fisher's exact test were performed and shown by *p*-values. The statistical significance level was set at less than 0.05. AG: acupuncture group, UG: Usual care group.

Table 7 mMRC

Dyspnea mMRC (U)	Baseline	After 12 weeks	After 52 weeks	Change from baseline to after 12 weeks	Mean difference 95% CI	Change from baseline to after 52 weeks	Mean difference 95% CI
UG	2.5 [0.8]	2.8 [1.0]	3.1 [1.0]	0.3 [0.7]	-1.1 [-1.5 to -0.7]	0.6 [0.7]	-1.5 [-2.0 to -1.0]
AG	2.7 [1.0]	1.9 [0.8]	1.8 [0.7]	-0.8 [0.5]		-0.9 [0.9]	

Values are mean [SD] unless otherwise stated.
The number of patients in each case was 20 in UG and 20 in AG.

Dyspnea during activities of daily living

The mMRC improved from 2.7 [1.0] at baseline to 1.9 [0.8] at week 12, to 1.8 [0.7] at week 52 in the AG. The difference in the mMRC in the AG (-0.8 [0.5] at week 12, -0.9 [0.9] at week 52) were statistically significant by t-test compared with that in the UG (0.3 [0.7] at week 12, 0.6 [0.7] at week 52) (mean difference -1.1, 95% CI -1.5 to -0.7 at week 12, -1.5 [-2.0 to -1.0] at week 52).

AG: Acupuncture group, UG: Usual care group, 95%CI: 95% confidence interval, mMRC: modified medical research council

Table 8 CAT

QOL CAT	Baseline	After 12 weeks	After 52 weeks	Change from baseline to after 12 weeks	Mean difference 95% CI	Change from baseline to after 52 weeks	Mean difference 95% CI
UG	17.7 [6.4]	16.9 [6.7]	19.6 [8.3]	-0.8 [3.2]	-6.9 [-9.9 to -3.8]	1.9 [5.7]	-11.4 [-15.8 to -6.9]
AG	18.8 [8.4]	11.2 [4.8]	9.4 [4.6]	-7.7 [6.0]		-9.5 [7.9]	

Values are mean [SD] unless otherwise stated.
The number of patients in each case was 20 in UG and 20 in AG.

CAT

The CAT improved from 18.8 [8.4] at baseline to 11.2 [4.8] at week 12, to 9.4 [4.6] at week 52 in the AG. The difference in the CAT in the AG (-7.7 [6.0] at week 12, -9.5 [7.9] at week 52) were statistically significant by t-test compared with that in the UG (-0.8 [3.2] at week 12, 1.9 [5.7] at week 52) (mean difference -6.9, 95% CI -9.9 to -3.8 at week 12, -11.4 [-15.8 to -6.9] at week 52).

AG: Acupuncture group, UG: Usual care group, 95%CI: 95% confidence interval, QOL: quality of life, CAT: COPD assessment test

Table 9 BODE index

Prognosis BODE index	Baseline	After 12 weeks	After 52 weeks	Change from baseline to after 12 weeks	Mean difference 95% CI	Change from baseline to after 52 weeks	Mean difference 95% CI
UG	4.4 [2.3]	4.6 [2.6]	5.5 [2.5]	0.2 [1.2]	-1.5 [-2.1 to -0.8]	1.1 [1.1]	-2.7 [-3.4 to -1.9]
AG	4.8 [2.1]	3.5 [1.7]	3.2 [1.9]	-1.3 [0.8]		-1.6 [1.2]	

Values are mean [SD] unless otherwise stated.
The number of patients in each case was 20 in UG and 20 in AG.

BODE index

The BODE index improved from 4.8 [2.1] at baseline to 3.5 [1.7] at week 12, to 3.2 [1.9] at week 52 in the AG. The difference in the BODE index in the AG (-1.3 [0.8] at week 12, -1.6 [1.2] at week 52) were statistically significant by t-test compared with that in the UG (0.2 [1.2] at week 12, 1.1 [1.1] at week 52) (mean difference -1.5, 95% CI -2.1 to -0.8 at week 12, -2.7 [-3.4 to -1.9] at week 52).

AG: Acupuncture group, UG: Usual care group, 95%CI: 95% confidence interval, BODE: BMI (B) degree of air flow obstruction (O) functional dyspnea (D) and exercise capacity (E)

Table 10 Impulse Oscillation System

Impulse Oscillation System	Baseline	After 12 weeks	After 52 weeks	Change from baseline to after 12 weeks	Mean difference 95% CI	Change from baseline to after 52 weeks	Mean difference 95% CI
**z5**							
UG	0.5 [0.2]	0.5 [0.2]	0.6 [0.2]	-0.0 [0.1]	-0.1 [-0.2 to 0.0]	0.1 [0.2]	-0.3 [-0.4 to -0.1]
AG	0.6 [0.2]	0.5 [0.2]	0.4 [0.1]	-0.1 [0.2]		-0.2 [0.2]	
**r5**							
UG	0.5 [0.1]	0.5 [0.2]	0.6 [0.2]	0.0 [0.1]	-0.2 [-0.3 to -0.1]	0.1 [0.1]	-0.3 [-0.4 to -0.2]
AG	0.5 [0.2]	0.3 [0.1]	0.3 [0.1]	-0.2 [0.1]		-0.2 [0.1]	
**r20**							
UG	0.3 [0.1]	0.3 [0.1]	0.3 [0.1]	0.00 [0.1]	-0.0 [-0.1 to 0.0]	0.1 [0.1]	-0.1 [-0.2 to -0.1]
AG	0.3 [0.1]	0.2 [0.1]	0.2 [0.1]	-0.0 [0.1]		-0.1 [0.1]	
**r5-r20**							
UG	0.2 [0.1]	0.2 [0.1]	0.2 [0.1]	0.0 [0.1]	-0.2 [-0.2 to -0.1]	0.1 [0.1]	-0.2 [-0.3 to -0.1]
AG	0.2 [0.1]	0.1 [0.1]	0.1 [0.1]	-0.1 [0.1]		-0.2 [0.1]	
**x5**							
UG	-0.3 [0.1]	-0.3 [0.2]	-0.3 [0.2]	0.0 [0.1]	0.1 [0.0 to 0.2]	-0.1 [0.1]	0.2 [0.1 to 0.3]
AG	-0.3 [0.2]	-0.2 [0.1]	-0.2 [0.1]	0.1 [0.2]		0.1 [0.2]	
**ax**							
UG	2.0 (1.5]	2.1 [1.7]	2.8 [2.0]	0.0 [0.9]	-0.9 [-1.8 to 0.1]	0.8 [1.3]	-2.0 [-3.0 to -0.9]
AG	2.3 [2.0]	1.5 [1.3]	1.1 [1.1]	-0.8 [1.9]		-1.2 [1.9]	

Values are mean [SD] unless otherwise stated.
The number of patients in each case was 20 in UG and 20 in AG.
At week 12, significant improvements in Impulse Oscillation System (r5, r5-r20 and x5) were found in the AG compared with the UG. At week 52, all Impulse Oscillation System evaluations showed significant improvements in the AG compared to the UG. AG: acupuncture group, UG: Usual care group.
